# Role of κ→λ light-chain constant-domain switch in the structure and functionality of A17 reactibody

**DOI:** 10.1107/S1399004713032446

**Published:** 2014-02-15

**Authors:** Natalia Ponomarenko, Spyros D. Chatziefthimiou, Inna Kurkova, Yuliana Mokrushina, Yuliana Mokrushina, Anastasiya Stepanova, Ivan Smirnov, Marat Avakyan, Tatyana Bobik, Azad Mamedov, Vladimir Mitkevich, Alexey Belogurov, Olga S. Fedorova, Michael Dubina, Andrey Golovin, Victor Lamzin, Alain Friboulet, Alexander A. Makarov, Matthias Wilmanns, Alexander Gabibov

**Affiliations:** a Shemyakin–Ovchinnikov Institute of Bioorganic Chemistry, Russian Academy of Sciences, ul. Miklukho-Maklaya 16/10, Moscow 117871, Russian Federation; b European Molecular Biology Laboratory, Hamburg Unit, c/o DESY, Notkestrasse 85, 22603 Hamburg, Germany; c Engelhardt Institute of Molecular Biology, Russian Academy of Sciences, Moscow 119991, Russian Federation; d Institute of Gene Biology, Moscow 117334, Russian Federation; e Institute of Chemical Biology and Fundamental Medicine, Siberian Branch, Russian Academy of Sciences, Novosibirsk 630090, Russian Federation; f St Petersburg Academic University, St Petersburg 194021, Russian Federation; g Lomonosov Moscow State University, Moscow 119991, Russian Federation; h Université de Technologie de Compiègne, Unité Mixte de Recherche 6022, Centre National de la Recherche Scientifique, 60205 Compiègne, France

**Keywords:** A17 reactibody, antibodies

## Abstract

The engineering of catalytic function in antibodies requires precise information on their structure. Here, results are presented that show how the antibody domain structure affects its functionality. The previously designed organophos­phate-metabolizing reactibody A17 has been re-engineered by replacing its constant κ light chain by the λ chain (A17λ), and the X-ray structure of A17λ has been determined at 1.95 Å resolution. It was found that compared with A17κ the active centre of A17λ is displaced, stabilized and made more rigid owing to interdomain interactions involving the CDR loops from the V_L_ and V_H_ domains. These V_L_/V_H_ domains also have lower mobility, as deduced from the atomic displacement parameters of the crystal structure. The antibody elbow angle is decreased to 126° compared with 138° in A17κ. These structural differences account for the subtle changes in catalytic efficiency and thermodynamic parameters determined with two organophosphate ligands, as well as in the affinity for peptide substrates selected from a combinatorial cyclic peptide library, between the A17κ and A17λ variants. The data presented will be of interest and relevance to researchers dealing with the design of antibodies with tailor-made functions.

## Abstract 2

Catalytic antibody variants with κ and λ light-chain constant domains show differences in their crystal structures which lead to subtle changes in catalytic efficiency and thermodynamic parameters as well as in their affinity for peptide substrates.

## 1. Introduction   

Exquisite specificity and high binding affinity, the hallmarks of the antibody (Ab) response, make Abs excellent tools for biotechnology and biomedical applications. During the past decade, achievements in the field of Ab engineering have markedly expanded the range of these applications. A number of engineering strategies have been applied to modify the functionality of therapeutic monoclonal Abs according to the requirements of the particular biological mechanism to be treated (Kaneko & Niwa, 2011 ▶; Klohn *et al.*, 2013 ▶; Lu *et al.*, 2012 ▶; Vincent & Zurini, 2012 ▶). The development of methods for the humanization and functional expression of Abs and their fragments, together with the emergence of powerful techniques for screening combinatorial libraries and the expansion of structure–function databases aided by refined X-­ray analysis, has opened unlimited opportunities for the engineering of Abs with tailor-made properties for specific applications. X-ray crystal structure analysis has played a crucial role in creating novel artificial biocatalysts and engineered Abs (Ekiert *et al.*, 2012 ▶; Golinelli-Pimpaneau *et al.*, 2000 ▶; Guenaga & Wyatt, 2012 ▶; Privett *et al.*, 2012 ▶; Turner *et al.*, 2002 ▶; Zheng *et al.*, 2004 ▶).

Antibody recognition of protein antigens is predominantly mediated by four to six complementarity-determining regions (CDRs), which are variable loops at the tip of each antigen-binding fragment (Fab). Fabs are composed of two polypeptide chains: heavy (H) and light (L). Each chain is folded into two distinct immunoglobulin (Ig) domains, the N-terminal variable domain (V_H_ or V_L_) and the C-terminal constant domain (C_H1_ or C_L_), with the amino-acid residues linking V_L_ to C_L_ and V_H_ to C_H1_ called the switch residues (J fragment, framework 4). The elbow angle, or elbow bend, defined as the angle between the pseudo-twofold axes relating V_L_ to V_H_ and C_L_ to C_H1_, is a highly variable parameter in antibodies, and its role in the antigen-binding capacity has been speculated on (Huber *et al.*, 1976 ▶; Landolfi *et al.*, 2001 ▶). It is still unknown how far the changes that a hapten induces in an Ab structure can extend. An analysis of Protein Data Bank (PDB) depositions has shown that in some cases significant differences exist between the elbow angles of liganded and unliganded Fabs (Stanfield, Zemla *et al.*, 2006 ▶; Stanfield, Gorny *et al.*, 2006 ▶). Dramatic changes in the domain structure of NC6.8 (an Ab directed against the compound NC174) caused by small ligand binding were revealed by X-ray structure analysis followed by various molecular-dynamics simulations (Guddat *et al.*, 1994 ▶, 1995 ▶; Sotriffer *et al.*, 2000 ▶). In particular, the elbow angle was shown to change by more than 30°. Mammalian Abs have two types of light chains, κ and λ, which are encoded by different chromosome loci. In general, λ-chain Abs have a less rigid conformation than κ-chain Abs, with the difference being reflected in the values of the elbow angle: about 195° in the former *versus* 125° in the latter (Stanfield, Zemla *et al.*, 2006 ▶). The apparent hyperflexibility of λ-chain Fabs may be owing to an insertion in their switch region, which usually consists of a glycine residue and hence can also provide more conformational freedom for their molecules.

**Figure 1:**
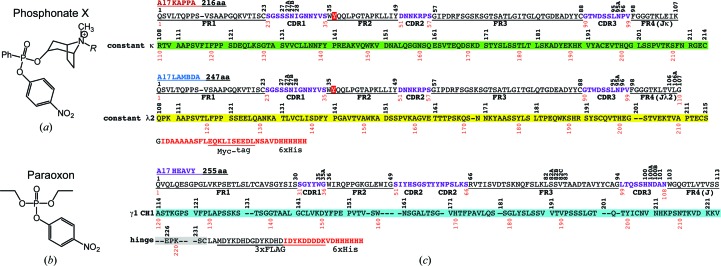
Chemical compounds and antibody chains used in this study. (*a*) *R* = H, *p*-nitrophenyl 8-methyl-8-azabicyclo[3.2.1]octylphenylphosphonate (phosphonate X); *R* = biotin, biotinylated phosphonate X (BtX). (*b*) *O*,*O*-Diethyl *O*-(4-nitrophenyl)phosphate (paraoxon). (*c*) Amino-acid sequences of A17 κ and λ light chains and the heavy chain. Residues are numbered using the Kabat system (superlinear) and sequential numbering (interlinear). Frameworks (FR1–FR4) are underlined; switch residues of the J segment are designated as FR4 according to Kabat; CDRs are coloured magenta; constant domains κC_L_, λC_L_ and C_H1_ are coloured green, yellow and blue, respectively.

Catalytic function is one of the most sophisticated features of Abs. Recently, the novel ‘reactibody’ approach has been developed, which is based on the chemical selection of bio­catalysts from a human semisynthetic Ab variable-fragment library followed by eukaryotic expression in a full-length Ab. This approach has been used to produce a novel organophosphate-metabolizing biocatalyst named the A17 reactibody (Reshetnyak *et al.*, 2007 ▶; Smirnov *et al.*, 2011 ▶). An important task is to develop effective antidotes against very toxic organophosphate compounds, including nerve agents and pesticides. Organophosphate poisoning is a serious clinical problem in rural regions of the developing world, causing the deaths of 200 000 people per year (Eddleston *et al.*, 2008 ▶). It has been shown that the A17 reactibody is capable of irreversibly binding phosphonate X (Fig. 1 ▶ *a*) and hydrolyzing the organophosphate pesticide paraoxon (Fig. 1 ▶ *b*) by covalent catalysis with rate-limiting dephosphorylation. The crystal structures of unmodified and phosphonylated Fabs of A17 have previously been solved at 1.5 and 1.36 Å resolution, respectively. Structural analysis combined with kinetic studies has provided an insight into certain mechanistic features of the reaction catalyzed by A17 (Smirnov *et al.*, 2011 ▶). In particular, the catalytic Tyr-L37 in this reactibody proved to be located in a deep active-site cavity, which is not typical of Abs in general. Some differences were observed in the catalytic efficiency of full-length A17 or its Fab fragment compared with its parent single-chain variable-fragment (scFv) molecule, which were possibly owing to structural stabilization of the active centre by additional constant domains. The full-length A17 reactibody contained an artificial light chain in which the λV_L_ domain was fused to κC_L_ through the κ switch region (Fig. 1 ▶ *c*), which possibly affected the functionality of the Ab. In the case of catalytic Abs, in which the active centre should have a rigid and precise structure to accomplish the catalytic function, cross-domain interaction effects can be extremely important. It has been shown that a change of the heavy-chain isotype (Sapparapu *et al.*, 2012 ▶) or Ab expression in the scFv format (Ponomarenko *et al.*, 2007 ▶) affects the catalytic activity or substrate specificity of Abs. It was reasonable to assume that the κ→λ switch would change the architecture of the binding pocket of the Ab molecule. To study the effects of such a switch, we produced a functionally active catalytic Ab with the constant λ light chain, thereby reconstructing the natural Ab structure.

In this study, we present data on the effect of a κ→λ light-chain switch on the structure and function of the A17 reactibody. The available high-resolution structural data allowed us to trace the changes caused by this switch in the active centre and antigen-binding site, and to observe how the interdomain interactions of the CDR loops narrow the cavity entrance, thereby forming a more rigid structure. A comparative structural analysis of the A17λ and A17κ variants showed that the κ→λ switch results in a decrease in the Ab elbow angle, in contrast to previously published data. It was somewhat unexpected that A17λ proved to have a rigid structure compared with A17κ, with its catalytic activity and affinity changing only slightly despite major structural modifications. Hopefully, the data presented below will contribute to the advancement of research on the design of antibodies with tailor-made functions.

## 2. Materials and methods   

### 2.1. Protein expression and purification   

Recombinant FabA17 containing κ or λ light-chain constant regions was produced in the methylotrophic yeast *Pichia pastoris* GS115 using the modified expression vector pPICZαA/Jk1 (Zakharov *et al.*, 2011 ▶) based on pPICZαA (Invitrogen, USA) (Supplementary Fig. S1^1^).

Expression constructs for HC_H_ (human Ab heavy chain containing the C_H1_ constant domain with hinge region) and A17 κ light chain (Zakharov *et al.*, 2011 ▶) and the constant region of human λ2 light chain with the J2 (joining) segment (Gabibov *et al.*, 2011 ▶) were prepared as described in the respective studies. To construct pPICZαA/Jλ2, the PCR-amplified DNA fragment corresponding to J2λ2 was digested with *Kpn*I and *Sac*II and ligated into pPICZαA/Jk1 at the appropriate restriction sites. V_L_A17 was amplified by PCR, digested with *Bsp*MI and *Spe*I, and cloned into pPICZαA/Jλ2 at the *Bsm*BI and *Avr*II restriction sites. All constructs were verified by DNA sequencing.

Procedures of electrocompetent cell preparation, electroporation of *P. pastoris* GS115 cells, Mut^+^ or Mut^s^ phenotype determination and selection on zeocin followed Invitrogen protocols. Analytical or large-scale expression of recombinant FabA17 was performed in cultures of BMGY and BMMY media according to the Invitrogen protocol. Methanol was added every 24 h after induction (up to 0.5%).

The culture medium was concentrated by ultrafiltration, equilibrated with 50 m*M* sodium phosphate buffer pH 8.0 containing 300 m*M* NaCl and purified on a Talon resin column (Clontech, USA). The eluted fraction was desalted against 50 m*M* sodium phosphate buffer pH 7.4 and separated by anion-exchange chromatography on a Mono Q column (Sigma) with salt-gradient elution (0–1 *M* NaCl in 50 m*M* sodium phosphate buffer pH 7.4). Fractions corresponding to Fabs were then purified on a Superdex 75 column (GE Healthcare, United States) equilibrated with 50 m*M* sodium phosphate buffer or 50 m*M* Tris–HCl buffer pH 7.4. The purity and identity of the eluted Fabs were tested by 12% SDS–PAGE with Coomassie staining and Western blot analysis. Horseradish peroxidase-conjugated anti-FLAG and anti-human light chain Abs (Sigma, USA) were used for detecting HC_H_ and C_L_, respectively.

### 2.2. Crystallization and data collection   

Crystals of FabA17λ were grown using the hanging-drop vapour-diffusion method by mixing equal volumes of protein (7 mg ml^−1^ in 50 m*M* Tris–HCl buffer pH 7.4) and precipitant solution [0.25 *M* ammonium sulfate and 20%(*w*/*v*) polyethylene glycol 5000 monomethyl ether in 0.1 *M* 2-(*N*-morpholino)ethanesulfonic acid (MES) pH 6.5]. Rod-shaped crystals of approximately 0.4 × 0.1 × 0.1 mm in size were obtained after 3–4 d and X-ray data were collected on EMBL beamline P14 at the PETRA III storage ring (DESY, Hamburg, Germany) at a wavelength of 1.2234 Å using a MAR CCD 225 mm detector. The data were collected at a cryogenic temperature of 100 K, and the mother-liquor solution supplemented with 20%(*v*/*v*) PEG 400 was used as a cryoprotectant. Images of 0.25° oscillation were collected over a total rotation of 85° using 2 s exposure per image. The diffraction data were indexed and integrated with *XDS* (Kabsch, 2010 ▶) and scaled using *SCALA* (Evans, 2006 ▶). The values of *I*/σ(*I*) and CC_1/2_ (Karplus & Diederichs, 2012 ▶) were used as a guide to determine the resolution cutoff (Table 1 ▶).

### 2.3. Structure solution and refinement   

The A17λ structure was solved by molecular replacement using *MOLREP* (Vagin & Teplyakov, 2010 ▶) with the heavy chain of the FabA17κ structure (PDB entry 2xza; Smirnov *et al.*, 2011 ▶) as a search model. Attempts to use the whole Fab structure as a model were unsuccessful, probably owing to the difference in the overall shape of the molecule.

The partial structure solution was followed by location of the V_L_ domain, which is identical in the λ and κ variants, and building of the rest of the Fab molecule was achieved using the *ARP*/*wARP* program (Langer *et al.*, 2008 ▶). A β-sheet of the C_L_ domain could not be built automatically and was added using several manual interventions with *Coot* (Emsley & Cowtan, 2004 ▶) and *PHENIX* (Afonine *et al.*, 2012 ▶), including simulated annealing. After completion of model building, refinement was carried out with *PHENIX* and *REFMAC*5 (Murshudov *et al.*, 2011 ▶).

**Figure 2:**
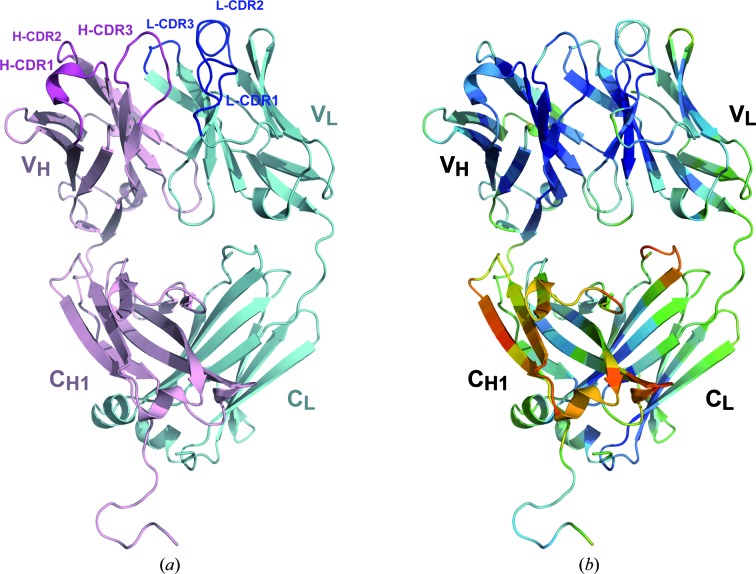
(*a*) The overall structure of the A17λ antibody. Heavy (V_H_/C_H1_) and light chains are shown in magenta and cyan, respectively. (*b*) The A17λ structure coloured according to the C^α^ atomic displacement parameters (ADP), with a colour transition from blue to red indicating increasing ADP values.

**Figure 3:**
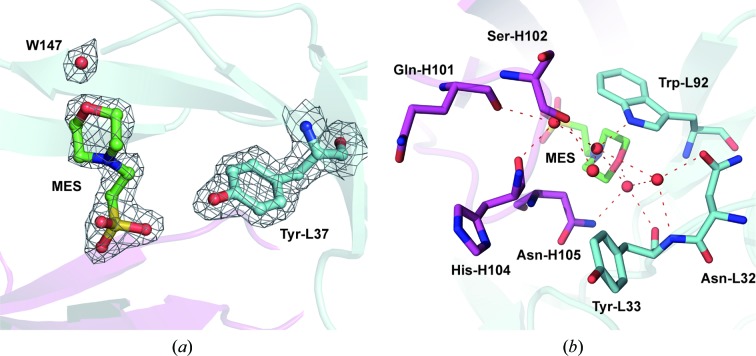
(*a*) 2*F*_o_ − *F*_c_ electron-density map contoured at the 1.5σ level above the mean (0.4 e Å^−3^) showing the MES molecule, water 147 and the catalytic Tyr-L36. Heavy and light chains are shown in magenta and cyan, respectively. (*b*) An extended hydrogen-bonding pattern involving the MES molecule and five intact water molecules located close to the A17λ active centre (red dashed lines).

Solvent molecules were located automatically using *PHENIX* and were confirmed by visual inspection; all of them were well defined in density. All located water molecules were refined with unit occupancy. The final model consisted of one Fab molecule (Fig. 2 ▶) with 445 residues and 310 water molecules. The positions of two N-terminal residues of the light chain could not be located in the electron density. Close to the active centre, there was a well resolved residual density for a MES molecule that was present in the crystallization condition and refined with occupancy value of 0.8 (Fig. 3 ▶). A stereochemical analysis of the structure using *PROCHECK* (Laskowski *et al.*, 1993 ▶) showed that 96.8% of the residues were in the most favoured regions of the Ramachandran plot and 3.2% were in favoured regions. The data were nominally collected to 1.89 Å resolution, but during processing the resolution was reduced to 1.95 Å. This moderate data truncation slightly improved the regions of poor electron density as well as the refinement statistics. The refinement was deemed to have converged at an *R* factor of 20.5% and an *R*_free_ of 25.5%. Data-collection and refinement statistics are presented in Table 1 ▶.

To check whether different search models or algorithms could affect the results of structure solution by molecular replacement, we also tried to use as search models individual domains from the A17κ structure or other reported Ab structures that share high homology with A17λ (*e.g.* PDB entries 3mly and 4evn; Jiang *et al.*, 2010 ▶; Lingwood *et al.*, 2012 ▶). Thus, solving the A17κ structure with *MOLREP*, we first located the V_H_ domain using the V_H_ domain of the 2xza or the 3mly structure as a model and then the C_H1_ domain of the same structures. Thereafter, the V_L_ and C_L_ domains were located using as models the homologous domains from the 2xza and 4evn structures, respectively. Similar results were obtained by first locating the domains of the light chain and then those of the heavy chain or first the two variable domains and then the constant domains of the Ab. By carrying out molecular replacement with *Phaser* (McCoy *et al.*, 2007 ▶), it was also possible to locate the four domains of the Fab molecule by using individual domains of 2xza or 3mly for V_H_ and C_H1_ and of 4evn for V_L_ and C_L_ as search ensembles. The same results were also obtained by altering the order of the search ensembles. Finally, using the *BALBES* pipeline (Long *et al.*, 2008 ▶) it was possible to individually locate the V_H_, C_H1_ and V_L_ domains of A17λ and finally to use *ARP*/*wARP* to build most of the C_L_ domain. All of the solutions were very similar, with r.m.s.d. values of less than 1 Å for C^α^ atoms after rigid-body refinement.

### 2.4. Molecular-dynamics simulation   

The GPU-accelerated *GROMACS* 4.6.3 software package (Pronk *et al.*, 2013 ▶) was used for the simulation and analysis of MD trajectories using the Amber ff99SB-ILDN force field (Lindorff-Larsen *et al.*, 2010 ▶). Explicit solvent simulations were performed at a temperature of 300 K with a time constant for coupling of 0.1 ps under the control of a velocity rescaling thermostat and isotropic constant-pressure boundary conditions under the control of the Berendsen algorithm of pressure coupling with a time constant of 5 ps and application of the particle mesh Ewald method for electrostatic interactions (PME). A triclinic box of TIP3P water molecules was added around the protein to a depth of 20 Å on each side of the solute. Charges were neutralized by the addition of chloride ions. Additional NaCl was added to the systems to a final concentration of 0.14 *M*. In each of the simulations, there were two temperature-coupling groups, the first consisting of protein and the second consisting of water with Na^+^ and Cl^−^ ions. The time step for integration in all simulations was 2 fs. Coordinates were written to output as a trajectory file every 10 ps and the total time of simulation was 250 ns. All simulations were performed on a Lomonosov supercomputer provided by the SRCC of Moscow State University. Analysis of the trajectories was also performed using the *GROMACS* 4.6 software package.

### 2.5. Evaluation of kinetic and thermodynamic parameters for the reactibody reactions   

Kinetic measurements were made as described by Reshetnyak *et al.* (2007 ▶) and Smirnov *et al.* (2011 ▶). Briefly, reactions of FabA17λ and FabA17κ (3–32 µ*M*) with phosphonate X or paraoxon (Figs. 1 ▶ *a* and 1 ▶ *b*) over a concentration range of 10–500 µ*M* were carried out in 0.1 *M* sodium phosphate buffer pH 7.4 at different temperatures. Reaction rates were determined from the changes in absorbance at 405 nm owing to *p*-nitrophenol formation, and the rate constants were calculated using a *p*-nitrophenol extinction coefficient ∊ of 12 300 *M* ^−1^ cm^−1^. Active Ab concentrations were extrapolated from the *A*_max_ at 405 nm in the presence of excess phosphonate X. Modifications of rate constants *k*_1_ were estimated by Kitz–Wilson analysis (for details, see Supporting Information §S1).

Stopped-flow measurements with fluorescence detection were made using an SX.18MV stopped-flow spectrometer (Applied Photophysics, England). All experiments were carried out in 20 m*M* sodium phosphate buffer pH 7.4 with 150 m*M* NaCl at different temperatures (280–293 K). The Trp fluorescence was excited at 290 nm and monitored at >320 nm. Each trace in the diagrams is the average of no fewer than four individual recordings. The concentration of Fabs in all experiments was 10 µ*M* and the concentrations of phosphonate and paraoxon were varied from 5 to 300 µ*M*. Kinetic parameters were calculated as described by Smirnov *et al.* (2011 ▶) using the *DynaFit* software (Kuzmic, 1996 ▶) (for details, see Supporting Information §S1).

Thermodynamic parameters (rate constants *k*_2_ and equilibrium constants *K*_d_; Supplementary Table S1) for the interaction of phosphonate X with A17 were determined.

### 2.6. Determination of denaturation temperature   

The denaturation temperatures (*T*_d_) of A17κ and A17λ were measured in a VP-DSC microcalorimeter (MicroCal, USA) in 0.5 ml cells at a heating rate of 1 K min^−1^ as described in Mitkevich *et al.* (2003 ▶). Test solutions contained 0.6–1.5 mg ml^−1^ protein in 50 m*M* sodium phosphate buffer pH 7.4. The accuracy of the measurements was ±0.1 K. To analyze functions of excess heat capacity, the *Origin-DSC* program package was used.

### 2.7. Phage library selection for binding the single-chain antibody scFvA17   

The random cyclic heptapeptide phage library C*X*_7_C was used (Koivunen *et al.*, 1994 ▶). The solid-phase selection procedures were performed according to Yribarren *et al.* (2003 ▶), with some modifications (for details, see Supporting Information §S2). The pool of phage-bound peptides selected after each round was tested for specificity toward scFvA17 by means of phage ELISA (Supplementary Fig. S2). DNA fragments encoding the peptides from 20 clones randomly taken after the fifth round were amplified by PCR and sequenced. As a result of amino-acid sequence alignment, two consensus sequences were identified: CRNPWGLTC (pep50) and CPNPWGLLC (pep54).

### 2.8. Peptide synthesis   

The peptides were synthesized by standard solid-phase Nα-Fmoc chemistry (for details, see Supporting Information §S3). Two selected peptides were obtained in cyclic (50C or 54C) and linear (50L or 54L) forms, each consisting of 18 amino acids. The two residues at the N-terminus belonged to the bacteriophage pIII protein and were followed by a peptide sequence flanked by two cysteines. Lysine in the C-terminal part (GAAGAEK), which is also found in the bacteriophage pIII protein, was conjugated with a biotin molecule. The final sequences of the peptides were as follows: NH_2_-GACRNP­WGLTCGAAGAEK(Biot)NH_2_ (50) and NH_2_-GACPNPW­GLLCGAAGAEK(Biot)NH_2_ (54).

### 2.9. Surface plasmon resonance (SPR) analysis   

The surface plasmon resonance experiments were performed using a Biacore T200 system (GE Healthcare) equipped with a research-grade SA sensor chip. Chemically synthesized peptides (oxidized pep50C and pep54C, reduced pep50L and pep54L and a control peptide) were immobilized according to the manufacturer’s protocol. Flow cell 1 was left blank as a reference surface. To collect kinetic binding data, A17κ, A17λ and control Abs were injected at a range of concentrations into the four flow cells at a flow rate of 10 µl min^−1^ at 298 K. Ab–peptide association and dissociation were each monitored for 300 s. The surfaces were regenerated by a 100 s injection of 100 m*M* glycine–HCl pH 2.0. Data were collected at a rate of 1 Hz and fitted with a 1:1 binding model using the affinity-analysis option available within the *BIA­evaluation* software.

## 3. Results and discussion   

### 3.1. Molecular organization of the A17 reactibody   

Organophosphate-metabolizing A17scFv with variable domains corresponding to IGHV4-b*/IGLV1-51* germline genes was selected from a human semisynthetic Ab variable fragment library using a covalent capture selection strategy (Reshetnyak *et al.*, 2007 ▶). To express full-length A17 reactibody in CHO cells, we used vectors permitting the production of a corresponding Ab with human constant domains of subclass IgG1/κ (Smirnov *et al.*, 2011 ▶). Some differences were observed in the catalytic efficiency of full-length A17 or its Fab fragment compared with the parent scFv molecule (Zakharov *et al.*, 2011 ▶). On one hand, these differences could result from structural stabilization of the active centre owing to the presence of additional constant domains. Crystallographic studies show that there is a close association between V_L_ and V_H_ and between C_L_ and C_H1_ in the Fab (Padlan, 1994 ▶). Typically, Fv shares similar antigen-binding properties with Fab. However, the relative orientation of V_L_ and V_H_ in Fv is obviously not necessarily the same as in the Fab because the stabilizing effect of the C_L_–C_H1_ module (observed in Fab) is absent in Fv (Narciso *et al.*, 2011 ▶). On the other hand, the full-length A17 reactibody contained the artificial light chain with the λV_L_ domain fused to κC_L_ through the κ switch region (Fig. 1 ▶ *c*) and this non-native domain could also affect the properties of Ab.

To solve this question, the natural λ light chain was constructed (Fig. 1 ▶ *c*) and recombinant FabA17 containing a κ or λ light-chain constant region was produced in the methylotrophic yeast *P. pastoris*.

### 3.2. Structure of the A17λ Fab reactibody and its comparison with the A17κ variant   

#### 3.2.1. Quality of the final model   

The A17λ crystals belonged to the tetragonal space group *P*4_1_2_1_2 and the model was refined to an *R* factor and *R*_free_ of 20.5 and 25.5%, respectively. The results showed that the asymmetric unit of the crystal contained one Fab molecule and four domains (V_L_, C_L_, V_H_ and C_H1_) with the canonical β-sandwich immuno­globulin fold (Fig. 2 ▶). The final model comprises residues 1–231 of the heavy chain and 3–216 of the light chain. Most of the structure, including the CDR regions, shows well defined electron density, except for the loops H 136–138, H 160–164 and L 170–173 (sequential numbering; for numbering according to Kabat, see Fig. 1 ▶ *c*).

#### 3.2.2. Comparison of the A17λ and A17κ structures   

As calculated by the *PISA* program (Krissinel & Henrick, 2007 ▶), the interface area between the heavy and light chains in A17λ is 1785 Å^2^ and there are 18 hydrogen bonds, one salt bridge and one disulfide bond (Supplementary Table S2). This interface is comparable to others reported for Fab structures, but is more extended than that in the A17κ structure (PDB entry 2xza; 1527 Å^2^ with 12 hydrogen bonds and one salt bridge).

**Figure 4:**
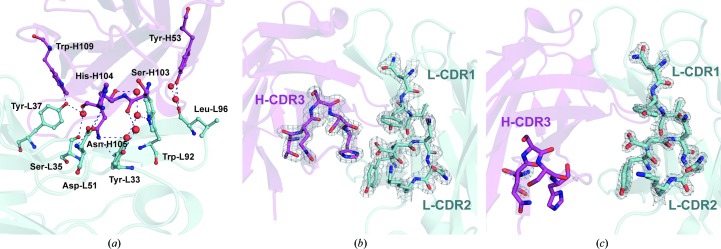
(*a*) Interdomain interactions between the CDR loops of A17λ and interactions involving catalytic Tyr-L37. Protein residues involved in these interactions are shown in ball-and-stick representation, water molecules are shown as spheres and hydrogen-bond interactions are shown as blue dashed lines. Here and in (*b*) and (*c*), heavy and light chains are shown in magenta and cyan, respectively. Tyr-L33 and Ser-L35 belong to L-CDR1, Asp-L51 to L-­CDR2, Trp-L92 and LeuL96 to L-CDR3, Tyr-H53 to H-CDR2, and Ser-H103, His-H104 and Asn-H105 to H-CDR3. (*b*) 2*F*_o_ − *F*_c_ electron-density map contoured at the 2σ level (0.5 e Å^−3^) above the mean for the CDR loop region of A17λ. (*c*) 2*F*_o_ − *F*_c_ electron-density map contoured at the 2σ level (0.5 e Å^−3^) above the mean for the CDR loop region of A17κ.

An important feature of the A17λ structure and the key difference between the two variants is a large interface between the two variable domains of the Fab molecule, where interactions take place between the CDR loops located close to the A17λ active centre, as well as Tyr-H53 from H-CDR2 (Fig. 4 ▶ *a*). They include strong direct interactions between residues of the H-CDR3 loop with L-CDR1 and L-CDR2, between H-CDR2 and L-CDR3 (Supplementary Table S2), as well as contacts *via* water molecules and a bound MES molecule. These interactions result in the displacement of these CDR loops, apart from H-CDR2, as follows from their structural alignment with the A17κ structure (r.m.s.d. of 2.4 Å for C^α^ atoms using *LSQKAB*; Kabsch *et al.*, 1976 ▶), and in the formation of a fairly rigid ensemble by the two variable domains, unlike in A17κ, where these domains do not interact directly. In contrast, the H-CDR1 loop (which does not take part in this interface) and the H-CDR2 loop, which lies farther away from the active centre (similar to H-CDR1), are well aligned in the two structures (r.m.s.d. of 0.9 Å using *LSQKAB*). The presence of a large interdomain interface in A17λ appears to provide stabilization of the CDR loops, as can be deduced from a comparison of their atomic displacement parameters (ADPs). Although the average ADP value for all protein atoms in the A17λ structure is higher than in A17κ (25.1 *versus* 21.5 Å^2^), the ADPs of the A17λ CDR loops are much lower (17.0 *versus* 25.4 Å^2^) (Supplementary Table S3). Furthermore, the electron density in this region is very well defined (Fig. 4 ▶ *b*), in contrast to the A17κ structure, in which the electron density of the CDR loops is relatively poor (Fig. 4 ▶ *c*). Comparison of the normalized ADP for residues of CDR loops for A17κ (PDB entry 2xza), phosphonylated A17κ (PDB entry 2xzc) and A17λ (PDB entry 3zl4) demonstrated that the ADP values for all CDR loops are generally higher for the two A17κ structures compared with A17λ, with no considerable dependence on the crystal contacts taking place in this loop (Supplementary Table S4 and Fig. S3). This in turn means that the active centre of the A17λ variant is more rigid than that of A17κ.

The reported A17λ structure is characterized by marked differences in the ADPs between the variable and constant domains of the chains. The average ADPs for all of the protein atoms in the V_H_ and V_L_ domains are 18.4 and 20.6 Å^2^, compared with 34.6 and 26.1 Å^2^ in the C_H1_ and C_L_ domains, respectively (Fig. 2 ▶ *b*). This is presumably owing to an extended interaction between the variable domains, with the constant domains being more mobile. In contrast, the A17κ variant shows an even distribution of ADP values, which average 19.2 and 19.2 Å^2^ for V_H_ and V_L_ and 17.3 and 19.3 Å^2^ for C_H1_ and C_L_, respectively. Certain differences in the ADPs between the variable and constant domains (with a slightly higher mobility for the constant domains) are not rare. In a survey of 200 high-resolution structures of unliganded Fab molecules deposited in the PDB, we found that the average ADPs for the C^α^ atoms of variable domains are almost identical, 29 Å^2^ for V_H_ and 28 Å^2^ for V_L_, with this parameter being slightly higher for the constant domains: 31 Å^2^ for both C_H1_ and C_L_ (Supplementary Fig. S4). Obviously, the corresponding differences in the case of A17λ are much greater.

**Figure 5:**
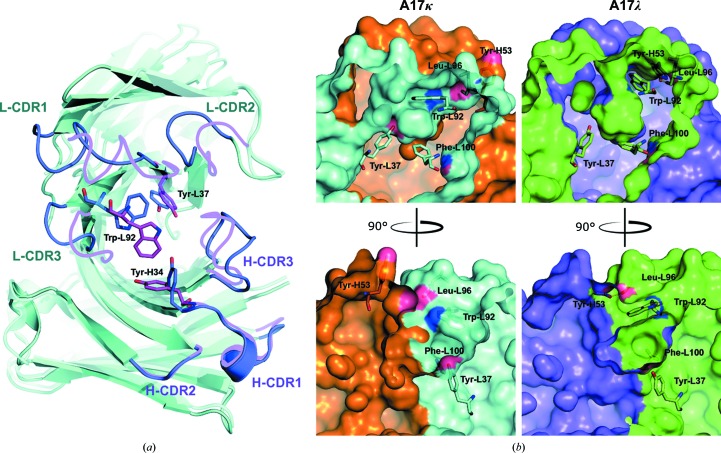
(*a*) Superposition of the A17λ and A17κ variable domains. A17λ CDR loops are shown in magenta and A17κ CDR loops are shown in blue. The flip of the Trp-L92 side chain is indicated. (*b*) The flipping of Trp-L92 provides the enlargement of the hydrophobic pocket surface (Trp-L92–Phe-L100) and the formation of a kind of lid above the cavity entrance. The light chain of A17κ is shown in cyan and the heavy chain is shown in brown; the light chain of A17λ is shown in green and the heavy chain in shown in magenta.

The active centre of A17λ structurally deviates from that of A17κ. The major difference is that the upper part of the A17λ active centre is shifted away from the light chain. This shifting of the L-CDR3 loop, which is mainly caused by the strong interaction between Tyr-H53 and Leu-L96, leads to the displacement of Trp-L92 by about 4 Å, its flipping and rotation (Fig. 5 ▶). This displacement of L-CDR3, and Trp-L92 in particular, which is probably facilitated by the crystal contacts or the presence of the MES molecule (Fig. 3 ▶ *b*), results in the enlargement of the hydrophobic pocket surface and the formation of a lid above the cavity entrance (Fig. 5 ▶ *b*). To estimate the level of involvement of the crystal contacts in the active-centre architecture, we performed an MD simulation of the behaviour of A17κ and A17λ in a dilute solution environment and without any neighbouring molecules, including MES for A17λ. In A17κ, displacement of the symmetry-related molecules by water leads to nonsignificant relaxation of the structure (protein backbone r.m.s.d. of 1.2 and 1.3 Å for the heavy and light chains, respectively). The same estimation performed for A17λ revealed noticeable changes in the structure (protein backbone r.m.s.d. of 2.2 and 2.7 Å for the heavy and light chains, respectively) and movement of L-­CDR3 and H-CDR3 followed by hydrogen-bond formation between the side chain of Trp-L92 and the main chain of Asn-H105 (Supplementary Fig. S5). It should be noted that this is not observed in the case of the A17κ MD structure during the entire simulation time; therefore, the rearrangement of A17λ mainly occurred owing to the removal of the MES molecule from the active site. The observations described above allow us to suggest that crystal contacts do not play a significant role in the structure of the A17κ active centre, but the impact of packing interactions for A17λ cannot be ruled out.

In the reported A17λ structure, the catalytic Tyr-L37 forms a hydrogen bond to Trp-H109, while a water molecule bridges it to Ser-L35 and Asn-H105, thereby forming a rigid entity (Fig. 4 ▶ *a*). In A17κ, Tyr-L37 does not directly interact with neighbouring residues but forms an extended hydrogen-bonding network through solvent molecules (Smirnov *et al.*, 2011 ▶). In the presence of co-crystallized MES, Tyr-H34 in A17λ is not directed towards Tyr-L37 but turns to form a hydrogen bond with Ser-H51. The positions of Tyr-L33 and Asn-H105 deviate from those in A17κ owing to a hydrogen-bonding pattern that is formed *via* one MES and five water molecules located close to the active centre. We think that this fairly extended hydrogen bonding (Fig. 3 ▶ *b*) stabilizes the protein and at the same time prevents the modification of A17λ by phosphonate X in crystals containing MES, in contrast to the crystal structure of A17κ, in which this modification is possible.

#### 3.2.3. Stability of the antibody variants   

To determine whether this extended hydrogen-bonding pattern at the active centre of A17λ and interdomain inter­action stabilize the Fab molecule, we measured the denaturation temperatures (*T*_d_) of κ and λ Fabs and their phosphonylated forms. The denaturation curves for the proteins had only one peak, indicating that the Fab molecules formed a single structural ensemble in all variants. The *T*_d_ values of the two λ variants were higher than those of the κ variants (339.8 *versus* 335.5 K for unmodified proteins and 347.1 *versus* 344.9 K for phosphonylated proteins, respectively).

These results indicate that the κ→λ switch leads to stabilization of the antibody molecule, possibly owing to the aforementioned interaction between the CDR loops. Furthermore, higher *T*_d_ values in the phosphonylated variants compared with the unmodified antibodies indicate that the phosphonylation stabilizes the antibody molecule.

#### 3.2.4. Structural alignment   

**Figure 6:**
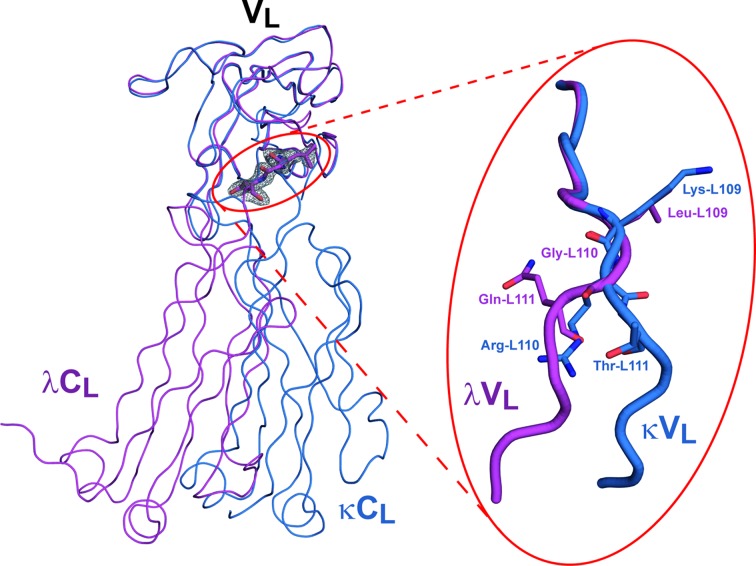
Structural superposition of the A17κ (blue) and A17λ (magenta) light chains on the V_L_ domain. The Gly-L110 insertion in A17λ accounts for the change in the V_L_–C_L_ orientation compared with that in A17κ.

Structure-based sequence alignment of λ and κ light chains revealed a high similarity between them. The variable domains of the light chain are identical (Supplementary Fig. S6), while the C_L_ domains and J regions connecting the V_L_ and C_L_ domains show only 42% sequence identity and 83% secondary-structure matching as calculated by the *PDBeFold* algorithm (Krissinel & Henrick, 2004 ▶). Alignment of the C^α^ atoms of the two structures using the *LSQKAB* algorithm resulted in r.m.s.d. estimates of 1.1 Å for the heavy chains and 4.1 Å for the light chains. Such a large structural deviation between κ and λ chains mainly appears to be owing to the J segment, especially to the Gly-L110 insertion in the λ variant, which changes the orientation of the C_L_ domain relative to V_L_ so that it forms a rotation angle of 29° with the latter (Fig. 6 ▶). In addition to the Gly-L110 insertion, the substitution of the positively charged Arg-L111 in A17κ by Gln-L111 in A17λ also appears to play a role in differentiation between the two light chains, since Gln-L111 strongly interacts with Tyr-L143, Val-L108 and Glu-L84. In A17κ, Arg-L111 is turned away from Tyr-L143, possibly because of steric effects, and forms a hydrogen bond with Asp-L172.

#### 3.2.5. Comparison of the elbow angles   

**Figure 7:**
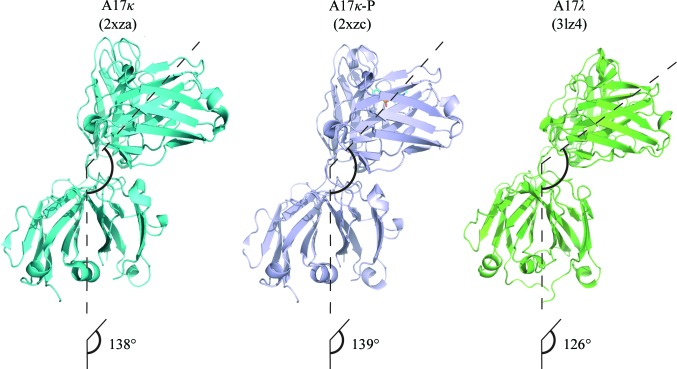
Elbow angles in A17 reactibody variants. A17κ-P is a phosphonylated variant.

The elbow angle of the FabA17λ molecule is 126°. This value is at the lower limit of the range previously recorded in Fab molecules (125–225°) and differs considerably from that characteristic of λ chain-type Fabs, which tend to adopt large elbow angles (Stanfield, Zemla *et al.*, 2006 ▶). Structural comparisons of A17λ with A17κ (PDB entry 2xza) and A17κ phosphonylated by phosphonate X (PDB entry 2xzc) allowed us to conclude that neither the κ→λ light-chain switch nor phosphonylation have any significant effect on the elbow angle (Fig. 7 ▶). As for the overall structure of the two constant domains (C_H1_ and C_L_) in the Fab molecule, the change in the C_L_ domain of A17λ resulted in some differences in its orientation relative to C_H1_, as follows from the comparison of the twist/tilt angles between the two domains (176/97° in A17λ *versus* 184/104° in A17κ) and in the alignment of these modules in the two structures (r.m.s.d. of 3.2 Å when aligned using the *LSQKAB* algorithm).

### 3.3. Functional characteristics of FabA17 reactibody variants with κ and λ light chains   

The newly obtained FabA17λ is a fully functional reactibody with a reactivity comparable to that of the κ variant. Comparison between A17 reactibodies with κ and λ light chains with respect to the steady-state kinetic parameters of their interaction with the phosphonate X molecule showed that *K*_d_ and *k*_2_ for A17λ were similar to those for A17k (Table 2 ▶). The slight difference in *K*_d_ indicates a certain divergence between these variants at the first (rapid) stage of ligand binding (see Scheme 1 in Supporting Information S§1).

To reveal the details of the reaction process, thermodynamic and pre-steady-state kinetic parameters were evaluated for A17κ and A17λ.

Thermodynamic parameters were calculated from the values of the *K*_d_ and *k*_2_ constants as a function of temperature (Supplementary Table S1 and Fig. S7). The energies of A17κ and A17λ modification by phosphonate X are comparable in terms of enthalpy and free energy; however, the reaction of A17λ with phosphonate X is more entropically favourable (Table 2 ▶).

The pre-steady-state kinetic analysis of interaction of A17λ with phosphonate X revealed two noncovalent binding stages, with the first rapid stage corresponding to the bimolecular interaction of the reactibody with the phosphonate ligand and the second stage involving induced-fit conformational changes of the reactibody. This is in agreement with previously reported data on the A17κ reactibody (Smirnov *et al.*, 2011 ▶). Comparison of kinetic parameters for A17κ and A17λ shows that the induced-fit stage is more rapid in the κ variant (Table 3 ▶). This may be owing to the less rigid structure of A17κ, with the entrance to its active centre being wider than in A17λ, where it is significantly narrowed because of inter­actions between the CDR loops. These conformational peculiarities provide more rapid and precise fitting of this reactibody to the phosphonate molecule.

An X-ray analysis of both A17 variants has shown that they have a well developed, deep active centre with the nucleophilic Tyr-L37 at the bottom of the cavity (15 Å from the surface of the molecule). A similar architecture of the active centre with Tyr-L37 has previously been described for the mouse catalytic Ab 13G5 (Heine *et al.*, 1998 ▶). This part of the Ab molecule is highly conserved and belongs to its structural core, which usually does not interact with antigens (Narciso *et al.*, 2012 ▶). This fact may account for the absence of marked differences between A17κ and A17λ in the kinetic parameters of their interaction with organophosphates. The observed structural divergence of CDRs loops can lead to changes in the antigen-binding properties of Ab molecules. Since A17 was selected as a biocatalyst using artificial chemical substrates, it was relevant to find out whether there is an epitope to which A17 is naturally intended to bind. As we failed to detect such an antigen among nucleic acids, lipids and polysaccharides (data not shown), we focused on epitopes of a protein nature. To address the question of the antigen specificity of A17κ and A17λ, we used a combinatorial approach. ‘Epitope mapping’ was performed by screening a phage-displayed cyclic heptapeptide library. To facilitate this procedure, scFvA17 was used for peptide-epitope selection. The scFv molecule consists of only the variable domains of the light and heavy chains connected through an (SG_4_)_2_SGGSAL linker; therefore, any effect from the constant domains was excluded. The scFvA17 proved to specifically bind two phage-displayed peptides, pep50 and pep54. It was found that the recombinant Fab reactibodies were also capable of binding the selected phage-displayed peptides pep50 and pep54, retaining this capacity after modification by phosphonate X (Supplementary Fig. S8). However, phosphonylation of the Fabs reduced the level of binding (Supplementary Fig. S8*a*), whereas such a modification of scFv significantly enhanced the ELISA signals (Supplementary Fig. S8*b*). The phosphonate ligand is completely buried in the active centre, being inaccessible for direct interaction with the peptides. Therefore, we suggest that covalent modification may have an indirect effect on the interaction of the antibody with the peptides.

To make a more accurate comparison of antigen-binding properties between the two A17 variants, we performed SPR analysis of reactibody binding with synthetic cyclic (pep50C and pep54C) and linear (pep50L and pep54L) peptides.

The affinity parameters determined from the sensorgrams are shown in Table 4 ▶ and Supplementary Fig. S9. Both A17κ and A17λ proved to have high affinity for the pep50L and pep50C peptides. However, A17κ was more active in binding either the linear or the cyclic form of pep50, which could be owing to its less rigid structure. In the case of pep54C, A17λ was more active than A17κ. A probable explanation is that the cyclic structure of pep54C fits better to the rigid A17λ antigen-binding site.

## 4. Conclusions   

Determination of the structure of the A17λ reactibody variant and its comparison with the previously reported structure of A17κ has provided an insight into changes in the structure–function relationship upon the κ→λ switch. It should be emphasized that the results presented above are based on direct comparison between the two light-chain variants of the same antibody A17, rather than on a statistical survey of structures reported for different antibodies. They show that an exchange of the light-chain constant domain produces an effect on the active-centre architecture, altering the overall shape of the light chain and consequently its orientation relative to the heavy chain. This alteration is also reflected in the interaction between the variable domains *via* their CDR loops, which leads to deformation of the cavity entrance and makes the binding pocket of A17λ different from that of A17κ; however, the impact of packing interactions cannot be formally ruled out. The elbow angle differs slightly between the two variants and is slightly reduced in A17λ compared with A17κ, in contrast to the data reported previously for antibodies with κ and λ light chains. As suggested previously (Stanfield, Zemla *et al.*, 2006 ▶), changes in elbow angles may simply serve to increase the flexibility of the Fab. Such changes could affect the reaction mechanism of the biocatalyst. Overall, the physicochemical (crystallographic, kinetic and thermodynamic) data and the results of artificial epitope mapping for both light-chain variants of A17 show that the replacement of the light-chain constant domain has an effect on the stability and antigen-binding properties of the reactibody, but not on its reactivity. In our opinion, the domain structure of an antibody molecule should be taken into account in the design of novel artificial biocatalysts.

## Supplementary Material

PDB reference: A17λ, 3zl4

Supporting Information.. DOI: 10.1107/S1399004713032446/wd5216sup1.pdf

## Figures and Tables

**Table 1:** Data-collection and refinement statistics

Data-collection statistics	
Source	MX beamline P14, PETRA III, EMBL/DESY
No. of images	380
Oscillation range ()	0.25
Space group	*P*4_1_2_1_2
Unit-cell parameters ()	*a* = *b* = 60.63, *c* = 279.64
Wavelength ()	1.2234
Resolution ()	25.01.95 (2.061.95)
*R*_merge_ (%)	9.6 (85.4)
*R*_r.i.m._ (%)	10.4 (92.0)
*R*_p.i.m._ (%)	3.8 (33.9)
CC_1/2_	99.7 (79.3)
*I*/(*I*)	15.6 (2.7)
Completeness (%)	99.9 (100)
Multiplicity	7.3 (7.3)
Estimated *B* factor from Wilson plot (^2^)	26.9
Refinement statistics	
Resolution range ()	25.01.95
No. of reflections used for *R*_free_ calculation	39334/1971
*R*_work_/*R*_free_ (%)	20.5/25.5
No. of atoms	
Protein	3326
Ligands	12
Solvent	310
*B* factors (^2^)	
Protein	24.8
V_H_	18.4
C_H_	34.6
V_L_	20.6
C_L_	26.1
Ligands	19.1
Solvent	27.8
Root-mean-square deviations	
Bond lengths ()	0.01
Bond angles ()	1.12
Ramachandran plot, residues in	
Most favoured regions	427 [96.8%]
Favoured regions	20 [3.2%]
Disallowed regions	0

**Table 2:** Kinetic parameters for the interaction of phosphonate X with and variants of A17

	Phosphonate X							Paraoxon
	*k*_2_ (min^1^)	*K*_d_ (*M*)	*k*_2_/*K*_d_ (*M* ^1^min^1^)	*G* (kcalmol^1^)	*H* (kcalmol^1^)	*S* (calmol^1^ K^1^)	*E*_a_ † (kcalmol^1^)	*k*_2_/*K*_d_ (*M* ^1^min^1^)
A17	0.24 0.03	(120 15) 10^6^	2000 500	6.5 0.1	4.1 0.3	4.0 1.2	12.5 1.3	1.2 0.5
A17	0.28 0.02	(130 15) 10^6^	2200 400	6.5 0.1	3.0 0.4	7.6 1.8	11.4 0.5	1.6 0.6

† *E*_a_ is the activation-energy parameter.

**Table 3:** Stopped-flow kinetic parameters of the interaction of phosphonate X with and variants of A17 The errors indicated are 1 SD.

	*k*_1_ (*M* ^1^s^1^)	*k*_1_ (s^1^)	*k*_2_ (s^1^)	*k*_2_ (s^1^)	*k*_obs1_	*k*_obs2_
A17	(6.6 0.5) 10^6^	460 35	46 7	150 20	530 90	130 30
A17	(17.5 0.8) 10^6^	480 35	65 10	90 12	580 90	80 20

**Table 4:** BiaCore affinity constants for interactions of A17 and A17 reactibodies with linear (50L and 54L) and cyclic (50C and 54C) peptides

	*K*_d_(50L) (10^6^ *M*)	*K*_d_(54L) (10^6^ *M*)	*K*_d_(50C) (10^6^ *M*)	*K*_d_(54C) (10^6^ *M*)
A17	1.3 0.1	25.0 5.0	0.9 0.1	25.7 0.5
A17	2.3 0.3	18.0 4.0	2.20 0.08	4.8 0.6

## Footnotes

1 Supporting information has been deposited in the IUCr electronic archive (Reference: WD5216).

## References

[bb1] Afonine, P. V., Grosse-Kunstleve, R. W., Echols, N., Headd, J. J., Moriarty, N. W., Mustyakimov, M., Terwilliger, T. C., Urzhumtsev, A., Zwart, P. H. & Adams, P. D. (2012). *Acta Cryst.* D**68**, 352–367. 10.1107/S0907444912001308 PMC3322595 22505256

[bb2] Eddleston, M., Buckley, N. A., Eyer, P. & Dawson, A. H. (2008). *Lancet*, **371**, 597–607. 10.1016/S0140-6736(07)61202-1 PMC2493390 17706760

[bb3] Ekiert, D. C. *et al.* (2012). *Nature (London)*, **489**, 526–532.

[bb4] Emsley, P. & Cowtan, K. (2004). *Acta Cryst.* D**60**, 2126–2132. 10.1107/S0907444904019158 15572765

[bb5] Evans, P. (2006). *Acta Cryst.* D**62**, 72–82. 10.1107/S0907444905036693 16369096

[bb6] Gabibov, A. G. *et al.* (2011). *FASEB J.* **25**, 4211–4221. 10.1096/fj.11-190769 21859892

[bb7] Golinelli-Pimpaneau, B., Goncalves, O., Dintinger, T., Blanchard, D., Knossow, M. & Tellier, C. (2000). *Proc. Natl Acad. Sci. USA*, **97**, 9892–9895. 10.1073/pnas.97.18.9892 PMC27617 10963661

[bb8] Guddat, L. W., Shan, L., Anchin, J. M., Linthicum, D. S. & Edmundson, A. B. (1994). *J. Mol. Biol.* **236**, 247–274. 10.1006/jmbi.1994.1133 7893280

[bb9] Guddat, L. W., Shan, L., Fan, Z.-C., Andersen, K. N., Rosauer, R., Linthicum, D. S. & Edmundson, A. B. (1995). *FASEB J.* **9**, 101–106. 10.1096/fasebj.9.1.7821748 7821748

[bb10] Guenaga, J. & Wyatt, R. T. (2012). *PLoS Pathog.* **8**, e1002806. 10.1371/journal.ppat.1002806 PMC3400562 22829767

[bb11] Heine, A., Stura, E. A., Yli-Kauhaluoma, J. T., Gao, C., Deng, Q., Beno, B. R., Houk, K. N., Janda, K. D. & Wilson, I. A. (1998). *Science*, **279**, 1934–1940. 10.1126/science.279.5358.1934 9506943

[bb12] Huber, R., Deisenhofer, J., Colman, P. M., Matsushima, M. & Palm, W. (1976). *Nature (London)*, **264**, 415–420. 10.1038/264415a0 1004567

[bb55] Jiang, X., Burke, V., Totrov, M., Williams, C., Cardozo, T., Gorny, M. K., Zolla-Pazner, S. & Kong, X.-P. (2010). *Nature Struct. Mol. Biol.* **17**, 955–961. 10.1038/nsmb.1861 20622876

[bb14] Kabsch, W. (2010). *Acta Cryst.* D**66**, 133–144. 10.1107/S0907444909047374 PMC2815666 20124693

[bb15] Kabsch, W., Kabsch, H. & Eisenberg, D. (1976). *J. Mol. Biol.* **100**, 283–291. 10.1016/s0022-2836(76)80064-2 3654

[bb16] Kaneko, E. & Niwa, R. (2011). *BioDrugs*, **25**, 1–11. 10.2165/11537830-000000000-00000 21033767

[bb17] Karplus, P. A. & Diederichs, K. (2012). *Science*, **336**, 1030–1033. 10.1126/science.1218231 PMC3457925 22628654

[bb18] Klohn, P. C., Wuellner, U., Zizlsperger, N., Zhou, Y., Tavares, D., Berger, S., Zettlitz, K. A., Proetzel, G., Yong, M., Begent, R. H. & Reichert, J. M. (2013). *MAbs*, **5**, 178–201. 10.4161/mabs.23655 PMC3893229 23575266

[bb19] Koivunen, E., Wang, B. & Ruoslahti, E. (1994). *J. Cell Biol.* **124**, 373–380. 10.1083/jcb.124.3.373 PMC2119941 7507494

[bb21] Krissinel, E. & Henrick, K. (2004). *Acta Cryst.* D**60**, 2256–2268. 10.1107/S0907444904026460 15572779

[bb22] Krissinel, E. & Henrick, K. (2007). *J. Mol. Biol.* **372**, 774–797. 10.1016/j.jmb.2007.05.022 17681537

[bb23] Kuzmic, P. (1996). *Anal. Biochem.* **237**, 260–273. 10.1006/abio.1996.0238 8660575

[bb24] Landolfi, N. F., Thakur, A. B., Fu, H., Vásquez, M., Queen, C. & Tsurushita, N. (2001). *J. Immunol.* **166**, 1748–1754. 10.4049/jimmunol.166.3.1748 11160220

[bb25] Langer, G. G., Cohen, S. X., Perrakis, A. & Lamzin, V. S. (2008). *Nature Protoc.* **3**, 1171–1179. 10.1038/nprot.2008.91 PMC2582149 18600222

[bb26] Laskowski, R. A., MacArthur, M. W., Moss, D. S. & Thornton, J. M. (1993). *J. Appl. Cryst.* **26**, 283–291.

[bb27] Lindorff-Larsen, K., Piana, S., Palmo, K., Maragakis, P., Klepeis, J. L., Dror, R. O. & Shaw, D. E. (2010). *Proteins*, **78**, 1950–1958. 10.1002/prot.22711 PMC2970904 20408171

[bb54] Lingwood, D., McTamney, P. M., Yassine, H. M., Whittle, J. R. R., Guo, X., Boyington, J. C., Wei, C.-J. & Nabel, G. J. (2012). *Nature (London)*, **489**, 566–570. 10.1038/nature11371 PMC7095019 22932267

[bb28] Long, F., Vagin, A. A., Young, P. & Murshudov, G. N. (2008). *Acta Cryst.* D**64**, 125–132. 10.1107/S0907444907050172 PMC2394813 18094476

[bb29] Lu, Z.-J., Deng, S.-J., Huang, D.-G., He, Y., Lei, M., Zhou, L. & Jin, P. (2012). *World J. Biol. Chem.* **3**, 187–196. 10.4331/wjbc.v3.i12.187 PMC3531614 23275803

[bb30] McCoy, A. J., Grosse-Kunstleve, R. W., Adams, P. D., Winn, M. D., Storoni, L. C. & Read, R. J. (2007). *J. Appl. Cryst.* **40**, 658–674. 10.1107/S0021889807021206 PMC2483472 19461840

[bb31] Mitkevich, V. A., Schulga, A. A., Ermolyuk, Y. S., Lobachov, V. M., Chekhov, V. O., Yakovlev, G. I., Hartley, R. W., Pace, C. N., Kirpichnikov, M. P. & Makarov, A. A. (2003). *Biophys. Chem.* **105**, 383–390. 10.1016/s0301-4622(03)00103-0 14499906

[bb32] Murshudov, G. N., Skubák, P., Lebedev, A. A., Pannu, N. S., Steiner, R. A., Nicholls, R. A., Winn, M. D., Long, F. & Vagin, A. A. (2011). *Acta Cryst.* D**67**, 355–367. 10.1107/S0907444911001314 PMC3069751 21460454

[bb33] Narciso, J. E. T., Uy, I. D. C., Cabang, A. B., Chavez, J. F. C., Pablo, J. L. B., Padilla-Concepcion, G. P. & Padlan, E. A. (2011). *New Biotechnol.* **28**, 435–447. 10.1016/j.nbt.2011.03.012 21477671

[bb34] Narciso, J. E. T., Uy, I. D. C., Cabang, A. B., Chavez, J. F. C., Pablo, J. L. B., Padilla-Concepcion, G. P. & Padlan, E. A. (2012). *Philipp. Sci. Lett.* **5**, 63–89.

[bb35] Padlan, E. A. (1994). *Mol. Immunol.* **31**, 169–217. 10.1016/0161-5890(94)90001-9 8114766

[bb36] Parmley, S. F. & Smith, G. P. (1988). *Gene*, **73**, 305–318. 10.1016/0378-1119(88)90495-7 3149606

[bb37] Ponomarenko, N. A., Pillet, D., Paon, M., Vorobiev, I. I., Smirnov, I. V., Adenier, H., Avalle, B., Kolesnikov, A. V., Kozyr, A. V., Thomas, D., Gabibov, A. G. & Friboulet, A. (2007). *Biochemistry*, **46**, 14598–14609. 10.1021/bi7013954 18020454

[bb38] Privett, H. K., Kiss, G., Lee, T. M., Blomberg, R., Chica, R. A., Thomas, L. M., Hilvert, D., Houk, K. N. & Mayo, S. L. (2012). *Proc. Natl Acad. Sci. USA*, **109**, 3790–3795. 10.1073/pnas.1118082108 PMC3309769 22357762

[bb39] Pronk, S., Páll, S., Schulz, R., Larsson, P., Bjelkmar, P., Apostolov, R., Shirts, M. R., Smith, J. C., Kasson, P. M., van der Spoel, D., Hess, B. & Lindahl, E. (2013). *Bioinformatics*, **29**, 845–854. 10.1093/bioinformatics/btt055 PMC3605599 23407358

[bb40] Reshetnyak, A. V., Armentano, M. F., Ponomarenko, N. A., Vizzuso, D., Durova, O. M., Ziganshin, R., Serebryakova, M., Govorun, V., Gololobov, G., Morse, H. C. III, Friboulet, A., Makker, S. P., Gabibov, A. G. & Tramontano, A. (2007). *J. Am. Chem. Soc.* **129**, 16175–16182. 10.1021/ja076528m PMC2527816 18044899

[bb42] Sapparapu, G., Planque, S., Mitsuda, Y., McLean, G., Nishiyama, Y. & Paul, S. (2012). *J. Biol. Chem.* **287**, 36096–36104. 10.1074/jbc.M112.401075 PMC3476277 22948159

[bb43] Smirnov, I. *et al.* (2011). *Proc. Natl Acad. Sci. USA*, **108**, 15954–15959.

[bb44] Sotriffer, C. A., Rode, B. M., Varga, J. M. & Liedl, K. R. (2000). *Biophys. J.* **79**, 614–628. 10.1016/S0006-3495(00)76320-X PMC1300962 10919996

[bb45] Stanfield, R. L., Gorny, M. K., Zolla-Pazner, S. & Wilson, I. A. (2006). *J. Virol.* **80**, 6093–6105. 10.1128/JVI.00205-06 PMC1472588 16731948

[bb46] Stanfield, R. L., Zemla, A., Wilson, I. A. & Rupp, B. (2006). *J. Mol. Biol.* **357**, 1566–1574. 10.1016/j.jmb.2006.01.023 16497332

[bb47] Turner, J. M., Larsen, N. A., Basran, A., Barbas, C. F. III, Bruce, N. C., Wilson, I. A. & Lerner, R. A. (2002). *Biochemistry*, **41**, 12297–12307. 10.1021/bi026131p 12369817

[bb48] Vagin, A. & Teplyakov, A. (2010). *Acta Cryst.* D**66**, 22–25. 10.1107/S0907444909042589 20057045

[bb49] Vincent, K. J. & Zurini, M. (2012). *Biotechnol. J.* **7**, 1444–1450. 10.1002/biot.201200250 23125076

[bb50] Yribarren, A. S., Thomas, D., Friboulet, A. & Avalle, B. (2003). *Eur. J. Biochem.* **270**, 2789–2795. 10.1046/j.1432-1033.2003.03651.x 12823549

[bb52] Zakharov, A. V., Smirnov, I. V., Serebryakova, M. V., Dronina, M. A., Kaznacheeva, A. V., Kurkova, I. N., Belogurov, A. A., Friboulet, A., Ponomarenko, N. A., Gabibov, A. G. & Bobik, T. V. (2011). *Mol. Biol.* **45**, 74–81. 21485500

[bb53] Zheng, L., Goddard, J. P., Baumann, U. & Reymond, J. L. (2004). *J. Mol. Biol.* **341**, 807–814. 10.1016/j.jmb.2004.06.014 15288788

