# Supplementary material for: Role of κ→λ light-chain constant-domain switch in the structure and functionality of A17 reactibody
Source: Acta Crystallogr D Biol Crystallogr. 2014 Feb 15;70(Pt 3):708–19. doi: 10.1107/S1399004713032446 (PMC3949517; doi:10.1107/S1399004713032446)
Supplement: Supplementary file 1 [file d-70-00708-sup1.pdf]

Supporting Information

S1. Evaluation of kinetic parameters for the reactibody reactions

Models for the recorded kinetic traces were simulated with the *DynaFit* program (Kuzmic, 1996) using an appropriate reaction mechanism with a single set of constants. The conformity of the fitted kinetic models to the experimental data was assessed by monitoring residuals against time for different scheme fits. The fitting of the experimental data to the one-step binding model for steady-state kinetic analysis or the two-step model for pre-steady-state kinetics (Scheme 1) yielded the values of the dissociation constant and rate constants of irreversible protein modification. The observed kinetic constants for two-step models was calculated using following formula:

k_{obs1}= $0.5\cdot(((k_1+k_{-1}+k_2+k_{-2}))+((k_1+k_{-1}+k_2+k_{-2})^2-4\cdot(k_1k_2+k_{-1}k_{-2}+k_1k_{-2}))^{1/2}$

k_{obs2}= $0.5\cdot(((k_1+k_{-1}+k_2+k_{-2}))+((k_1+k_{-1}+k_2+k_{-2})^2-4\cdot(k_1k_2+k_{-1}k_{-2}+k_1k_{-2}))^{1/2}$

The concentration of phosphonate X was determined as if it was represented by the P(R) enantiomer alone.

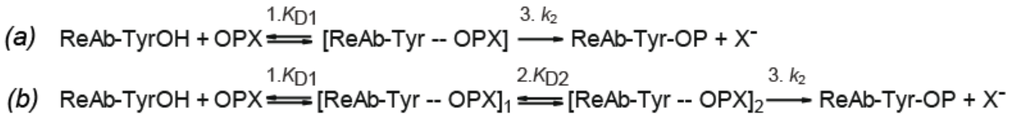

Scheme S1. (a) One-step reaction model and (b) induced fit reaction model of phosphonate X covalent binding by reactibody A17. Designations: ReAb-TyrOH, antibody; OPX, organophosphate compound; ReAb-Tyr–OPX, noncovalent antibody–substrate complex; ReAb-Tyr-OP, phosphonylated antibody; X, leaving group.

Paraoxon was expected to interact with the reactibody in a more complicated way, with covalent complex formation being followed by its hydrolysis. Consequently, corresponding Scheme 2 was obtained by adding one irreversible hydrolysis stage to Scheme 1. Its first step reflects substrate binding and formation of the catalytically active complex (Ab–OPX), and the final (irreversible) step pertains to the irreversible reaction of the modification. Thus, the final step of Scheme 2 most likely corresponds to the hydrolysis of the phosphonylated reactibody.

The kinetic curves for the interaction of A17 variants with paraoxon were fitted according to Scheme 2.

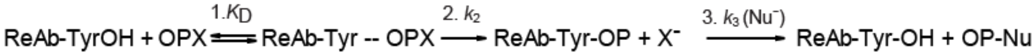

Scheme S2. Scheme of paraoxon hydrolysis by reactibody A17. For designations, see Scheme 1.

Thermodynamic parameters for phosphonate X interaction with A17 were determined using rate constants k_2 and equilibrium constants K_D (Table S1). The ΔH and ΔS values were calculated by the formula $\ln(K_A) = \Delta S/R + \Delta H/RT$. The Gibbs free energies ΔG at 298K were calculated as $\Delta G = \Delta H + T\Delta S$. Activation energy ($E_a$) was calculated from the Arrhenius equation: $\ln(k_2) = -E_a/RT + \ln(A)$.

S2. Phage library selection for binding scFvA17

Selection procedures were performed as described (Yribarren *et al.*, 2003), with appropriate modifications.

S2.1. Amplification and purification of phage peptide libraries

The random cyclic heptapeptide library (named CX7C) was kindly provided by E. Koivunen, Dept of Biosciences, Division of Biochemistry, University of Helsinki, Finland (Koivunen *et al.*, 1994). The library was amplified by infecting mid-log phase *Escherichia coli* cells with 3×10^{10} phage transforming units (TU) at 310K for 30 min, without shaking,. The infected bacteria were then grown overnight in 1 L Luria–Bertani medium containing 20 μg/mL tetracycline at 310K, on a shaker. The amplified phages were precipitated from the culture medium by adding 20% (v/v) of 20% polyethylene glycol 8000 in 2.5 M NaCl (PEG/NaCl) and incubating the mixture overnight at 277K. Phages were then pelleted by centrifugation at 10 000 *g* for 45 min at 277K, suspended in 10 mL of deionized water, filtered through a 0.45 μm filter unit (Millipore), and precipitated again with PEG/NaCl for 1 h at 277K. The library was finally recovered in 1 mL of Tris-buffered saline (TBS) pH 7.5 containing 50 mM Tris-HCl and 150 mM NaCl and stored at 253K. Titration of the library was performed by infecting mid-log phage *E. coli* cells with phage dilutions for 30 min at 310K, without shaking, and plating 100-μL aliquots on solid Luria–Bertani agar plates containing 20 μg/mL tetracycline. The plates were incubated overnight at 310K, and visible clones were then counted.

S2.2. Solid phase selection procedures

Selection was performed in 96-well MaxiSorp plates (Nunc, Denmark) by a panning method modified from Parmley & Smith (1988). Unless otherwise specified, each step of the procedure was followed by plate washing in three portions of TBS with 0.1% Tween 20 (TTBS) and incubation at 310K for 1 h without shaking. The plates were coated with 100 μL of single-chain scFvA17 antibody, 2–10 μg/mL in TBS (Reshetnyak *et al.*, 2007). The wells were then saturated with 100 μL of 2% milk in TBS (MTBS, w/v). Phage solution (5×10^{10}–2.5×10^{11} TU) was preincubated with MTBS (10 min at 310K) to avoid the binding of phage-displayed peptides to milk proteins, transferred into the wells, and incubated for 2 h at 293K. This step was followed by washing in 10 portions of TTBS (for at least 5 min in each). The bound phages were eluted from the plates by 10-min incubation at room temperature in 0.1 M HCl adjusted to pH 2.2 with solid glycine. The collected phage solution was brought to a neutral pH by adding 11% of 2 M Tris. The enrichment of the initial library in phage specific to scFvA17 was monitored by phage titration before and after each round of selection and expressed as the ratio of TU at round n to TU at round $n + 1$. After each round of selection, eluted phages were amplified by infecting 20 mL of mid-log phase bacteria, as described above, and then used for the next round.

The pool of page-displayed peptides selected at each round was tested for affinity to scFvA17 by phage ELISA (Supporting Fig. S2).

S2.3. Phage ELISA

Unless otherwise specified, the wells were filled with 100 μL of a given solution and the plate was incubated for 1 h at 310K, with each step being followed by washing in three portions of TTBS. MaxiSorp plates were coated with 1-μg aliquots of scFvA17, FabA17κ, FabA17λ, or their variants modified by phosphonate X in 0.1 M carbonate buffer pH 8.6. The wells were saturated with MTBS. A variable amount of phages (5×10^7 to 5×10^{10} TU per well) was then added and incubated in the presence of MTBS at room temperature for 2 h. After washing in 10 portions of TTBS, bound phages were detected using HRP/anti-M13 Monoclonal Conjugate (Amersham Pharmacia Biotech, England) diluted 1:5000 and subsequent reaction with 3,3',5,5'-

tetramethylbenzidine (TMB) for 15 min at room temperature. The reaction was stopped by adding 2M sulfuric acid, and the results were immediately quantified by measuring OD_{450} nm.

S2.4. Phage DNA sequencing

Oligonucleotides were purchased from Sintol (Moscow, Russia). The peptide-encoding DNA inserts from individual selected clones were amplified by PCR in an Eppendorf thermocycler (predenaturation at 367K for 10 min followed by 35 cycles of denaturation at 367K for 1 min, annealing at 327K for 1 min, and elongation at 345K for 1.5 min). Taq DNA polymerase was from New England Biolabs (United States). Amplification was performed with forward primer AB 348 (5'-TTAGCAAAACCTCATACAGAA-3') and reverse primer AB 349 (5'-GATGCTGTCTTTCGCTGCTGAG-3'). For sequencing, forward primer M13 (5'-ATTCACCTCGAAAGCAAGCTG-3') was used.

S3. Peptide synthesis

Two selected peptides were synthesized in cyclic and linear forms, each consisting of 18 amino acids. Two residues at the N-terminus belonged to bacteriophage pIII protein and were followed by a peptide sequence flanked by two cysteines. Lysine of the C-terminal part GAAGAEK was conjugated with a biotin molecule. The final sequences of the peptides were as follows: NH2-GACRNPWGLTCGAAGAEK(Biot)NH2 (pep50) and NH2-GACPNPWGLLCGAAGAEK(Biot)NH2 (pep54). The peptides were prepared by standard solid-phase Nα-Fmoc chemistry using HBTU/1-hydroxybenzotriazole carboxy-group activation on 4-(2',4'-Dimethoxyphenyl-Fmoc-aminomethyl)-phenoxy (rink-amide) resin. First, Fmoc-Lys(Mtt)-OH was coupled to rink-amide resin using the standard coupling protocol. This was followed by Mtt deprotection by treatment with 1.8% (v/v) TFA in DCM for 3 min at room temperature, which was repeated nine times using 10 mL of the solvent per gram resin, according to Li & Elbert (2002). Subsequent biotinylation of ε-NH2-group was performed by treating Fmoc-Lys(ε -NH2)-resin with a solution of 1.5 moleq of Biotin-ONp (300 mM) and 0.6 moleq of HOBt in NMP for 1 h. Other α-Fmoc-protected amino acids were coupled to the peptidyl-polymer by a standard procedure. Peptides were cleaved from the resin and deprotected by treatment with a trifluoroacetic acid–ethandithiol–water–triisopropylsilane mixture (94:2.5:2.5:1) for 2.5 h (1 ml mixture per 100 mg resin). Linear peptides were purified by

preparative HPLC. The purity and fidelity of the peptides were confirmed by RP–HPLC (purity ~98%) and mass spectrometry. For intramolecular cyclization, the HPLC-purified linear peptides were dissolved to a concentration of about 100 µM (0.2 mg/ml) in 100 mM NaHCO3 pH 7.5, and 0.2 volumes of DMSO were added to the solution. The process of cyclization was monitored by HPLC. The peak corresponding to the linear peptide was undetectable after 12 h of reaction. Resulting cyclic peptides were again purified by RP-HPLC, and the results were verified by RP-HPLC (purity ~99%) and mass spectrometry.

Table S1. Steady-state kinetic analysis of phosphonate X and paraoxon interactions with A17κ and A17λ reactibodies

		A17κ	A17λ
T, K	Kinetic parameters	Phosphonate X	
283	k_2, min^{-1}	0.07 ± 0.01	0.10 ± 0.02
	K_D, $M^{-1} \times 10^{-6}$	80 ± 5	110 ± 5
	k_2/K_D, $M^{-1}\ min^{-1}$	875 ± 180	909 ± 223
288	k_2, min^{-1}	0.13 ± 0.02	0.15 ± 0.03
	K_D, $M^{-1} \times 10^{-6}$	130 ± 10	145 ± 5
	k_2/K_D, $M^{-1}\ min^{-1}$	1000 ± 231	1034 ± 243
293	k_2, min^{-1}	0.18 ± 0.02	0.23 ± 0.03
	K_D, $M^{-1}x10^{-6}$	130 ± 5	170 ± 20
	k_2/K_D, $M^{-1}min^{-1}$	1385 ± 207	1353 ± 336
298	k_2, min^{-1}	0.24 ± 0.03	0.28 ± 0.02
	K_D, $M^{-1}x10^{-6}$	120 ± 15	130 ± 15
	k_2/K_D, $M^{-1}\ min^{-1}$	2000 ± 500	2154 ± 402
303	k_2, min^{-1}	0.32 ± 0.03	0.41 ± 0.03
	K_D, $M^{-1} \times 10^{-6}$	150 ± 15	180 ± 20
	k_2/K_D, $M^{-1}\ min^{-1}$	2133 ± 413	2278 ± 420
		Paraoxon	
298	k_2/K_D, $M^{-1}\ min^{-1}$	1.15 ± 0.52	1.63 ± 0.65

Table S2. Interactions between the light and heavy chains of A17λ. Interactions between the CDR loops are shown in bold type.

Light Chain		Heavy chain
	**Hydrogen bonds (Å)**	
Q39 [NE2]	2.89	Q40 [OE1]
Q39 [NE2]	3.45	Y95 [OH]
D51 [N]	**2.70**	N105 [OD1]
Y180 [OH]	2.87	S185 [OG]
G216 [N]	3.49	C222 [SG]
Y88 [OH]	3.24	Q40 [NE2]
Q39 [OE1]	3.04	Q40 [NE2]
L96 [O]	**3.24**	Y53 [OH]
D51 [OD2]	**2.59**	H104 [ND1]
D51 [OD1]	**3.14**	N105 [N]
Y33 [O]	**3.43**	N105 [ND2]
Y37 [OH]	2.98	W109 [NE1]
T208 [O]	2.78	K135 [NZ]
E127 [OE2]	2.71	K149 [NZ]
E163 [OE1]	3.35	S178 [N]
Y180 [OH]	3.23	S185 [N]
E126 [OE1]	2.79	K215 [NZ]
E213 [O]	3.11	K220 [NZ]
	**Salt bridge**	
E126 [OE2]	2.95	K215 [NZ]
	**Disulfide bond (Å)**	
C214 [SG]	2.02	C222 [SG]

Table S3. Comparison of ADP for CDR regions of A17κ and A17λ reactibodies.

CDR loop	ADP (Å^2)	
	**A17κ**	**A17λ**
**H-CDR1**	23.1	15.4
**H-CDR2**	20.0	17.8
**H-CDR3**	32.2	15.9
**L-CDR1**	23.6	19.6
**L-CDR2**	20.5	13.7
**L-CDR3**	22.9	18.8

Table S4. Crystal contacts of CDR loops in A17 antibody variants.

Residue 1	Distance (Å)	Residue 2	Symmetry operator
		A17κ (2XZA)	
H:Y59(OH)	2.73	L:R213(O)	x-1,y,z-1
H:S55(OG)	2.67	L:G214(O)	x-1,y,z-1
L:S95(O)	2.76	L:K192(NZ)	x-1,y,z-1
L:L96(O)	3.04	L:K192(NZ)	x-1,y,z-1
L:N97(OD1)	3.19	L:K192(NZ)	x-1,y,z-1
		A17λ	
H:A224(N)	2.92	L:N53(OD1)-	y+1/2,x-1/2,z+1/4
H:Y227(OH)	3.31	L:R55(O)	y+1/2,x-1/2,z+1/4
H:C222(O)	2.84	L:N52(ND2)	y+1/2,x-1/2,z+1/4
H:A224(O)	3.1	L:N53(ND2)	y+1/2,x-1/2,z+1/4
H:Y227(O)	3.39	L:N53(ND2)	y+1/2,x-1/2,z+1/4
H:K228(O)	3.28	L:N53(ND2)	y+1/2,x-1/2,z+1/4
L:S155(OG)	3.34	H:Y59(OH)	-y+1/2,x-1/2,z+1/4
L:D154(OD2)	2.59	H:S57(OG)	-y+1/2,x-1/2,z+1/4
L:D154(OD2)	2.7	H:S55(OG)	-y+1/2,x-1/2,z+1/4
L:S190(O)	3.25	H:S55(N)	-y+1/2,x-1/2,z+1/4
L:H191(O)	2.74	H:Y33(OH)	-y+1/2,x-1/2,z+1/4
L:R192(NH1)	3.05	L:S95(O)	-y+1/2,x-1/2,z+1/4
L:C214(N)	2.87	L:N31(O)	-y+1/2,x-1/2,z+1/4
L:S26(N)	3.09	H:S15(OG)	-x+1/2,y-1/2,-z+1/4
L:G24(O)	2.99	H:S85(OG)	-x+1/2,y-1/2,-z+1/4
L:D61(OD2)	2.64	H:S31(OG)	-x+3/2,y-1/2,-z+1/4

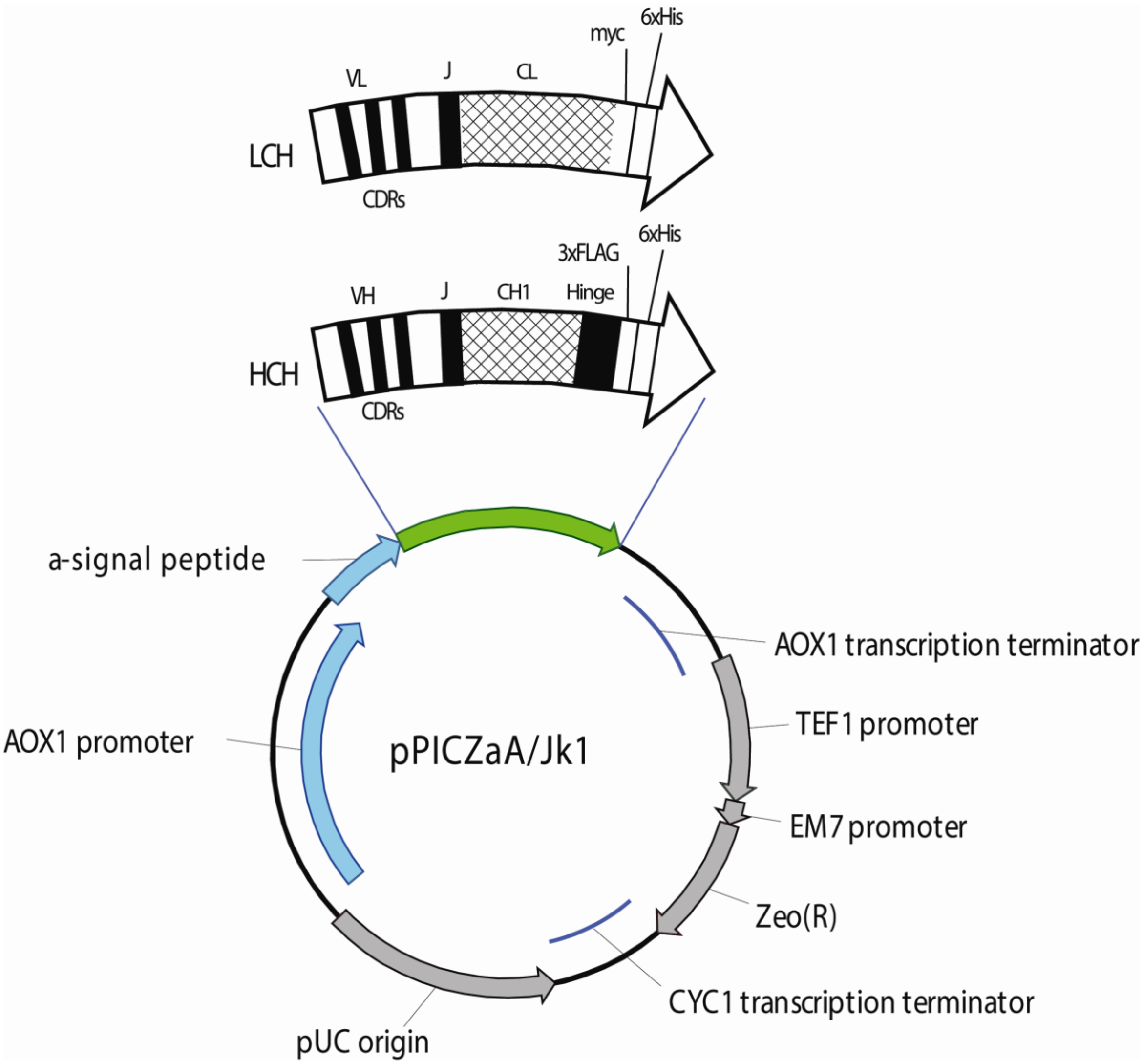

Figure S1. Genetic constructs for FabA17 expression.

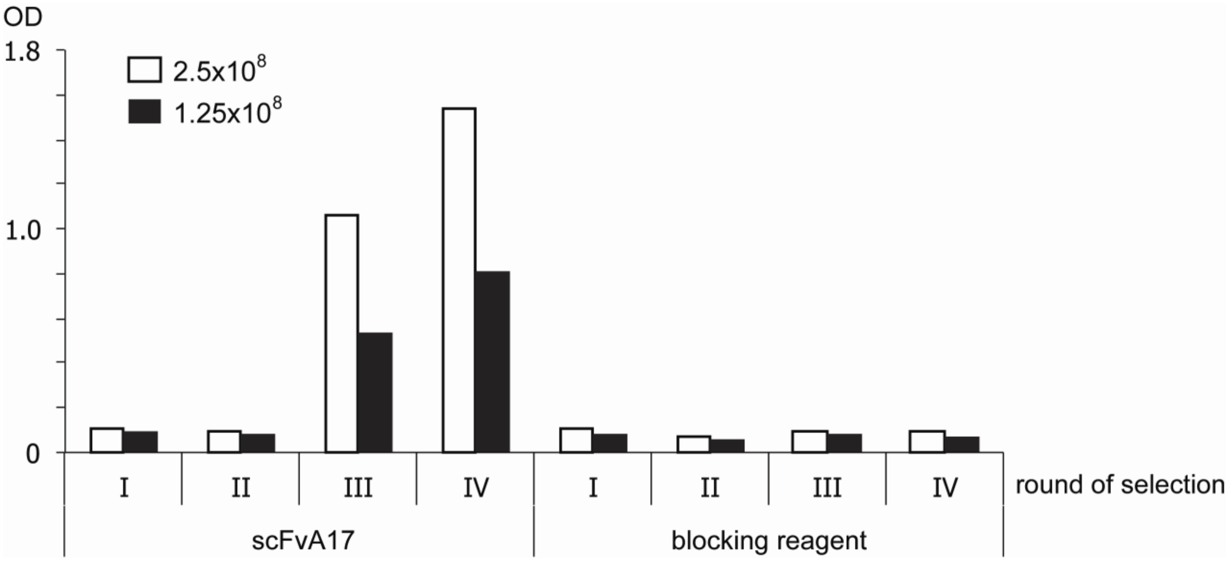

Figure S2. Phage ELISA of CX7C library enriched at each round of selection. The background signal is from the plate not coated with scFvA17 antibody.

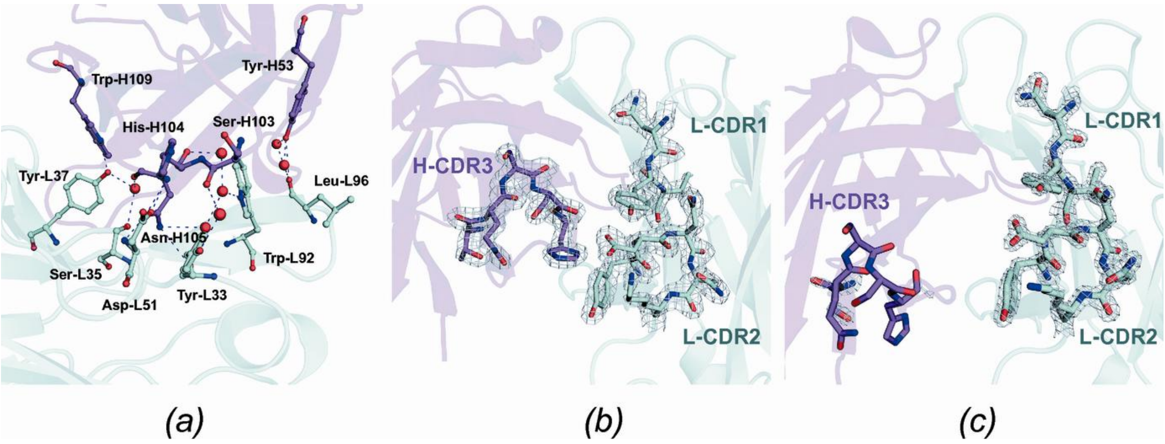

Figure S3. Normalized ADP values (ADP for the Cα atoms / Wilson B-factor) and crystal contacts for the residues of the CDR loops of the A17λ and A17κ structures (* for hydrogen bonds, ◊ for interfacing residues).

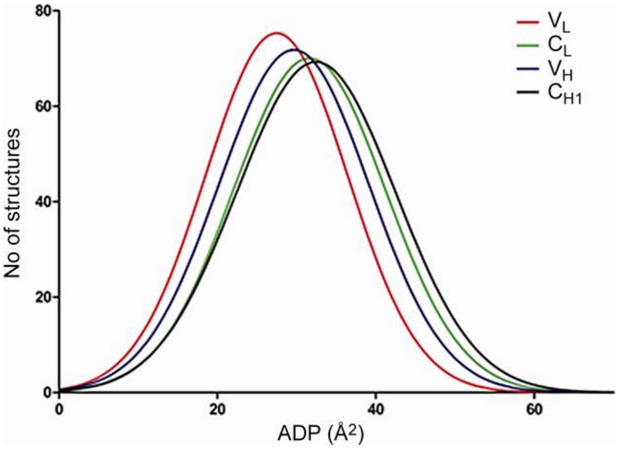

Figure S4. ADP distribution of the constant and variable domains of 200 high resolution unliganded Fab structures Å^2.

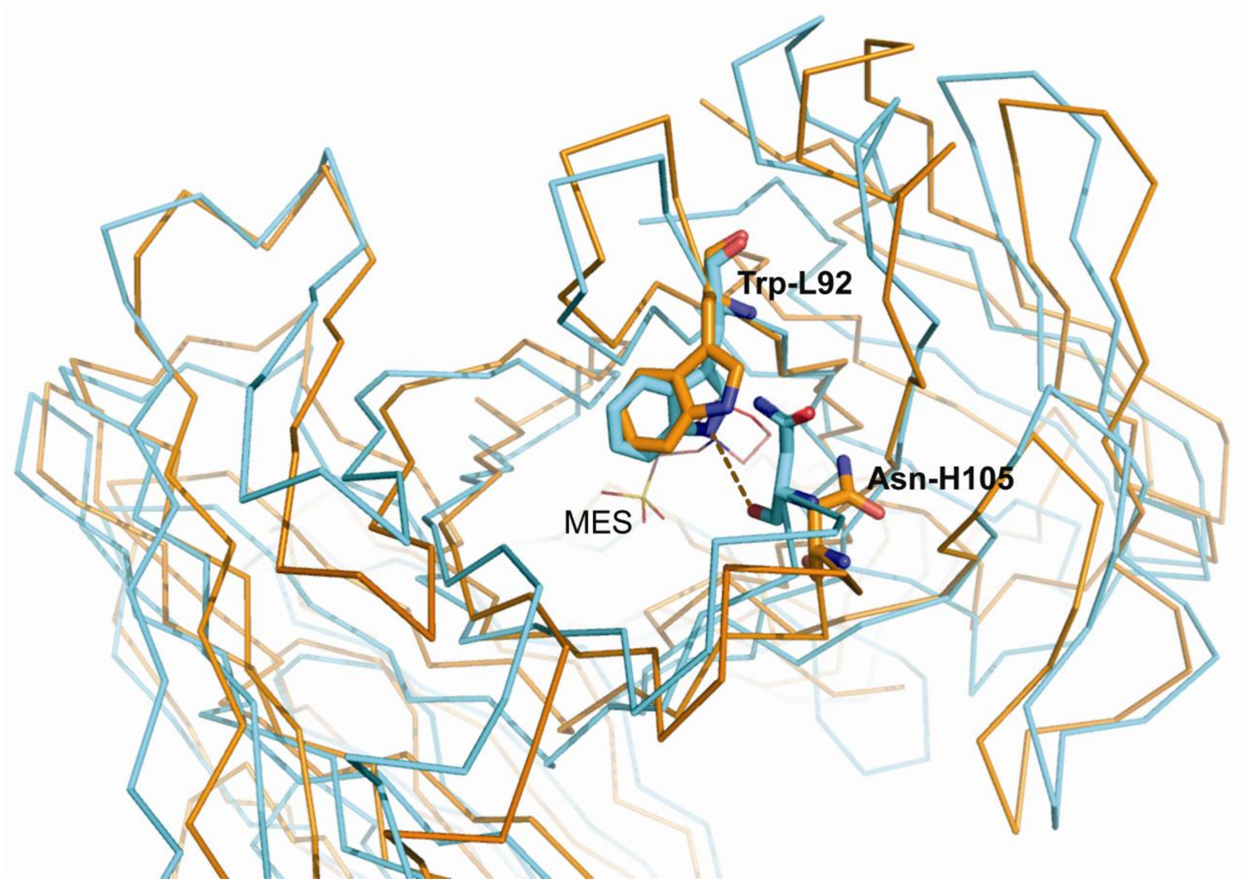

Figure S5. Superposition of the X-ray (orange) and MD (cyan) of the A17λ active center. The MD simulation reveals movement of L-CDR3 and H-CDR3 followed by H-bond formation between side chain of Trp-L92 and main chain of Asn-H105.

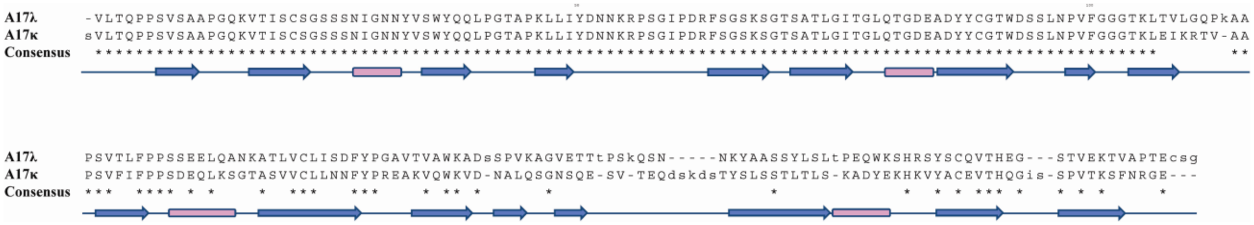

Figure S6. Structure-based sequence alignment of κ and λ light chains. Locations of secondary structural elements are indicated at the bottom (α-helices and β-strands are shown as arrows and cylinders, respectively)

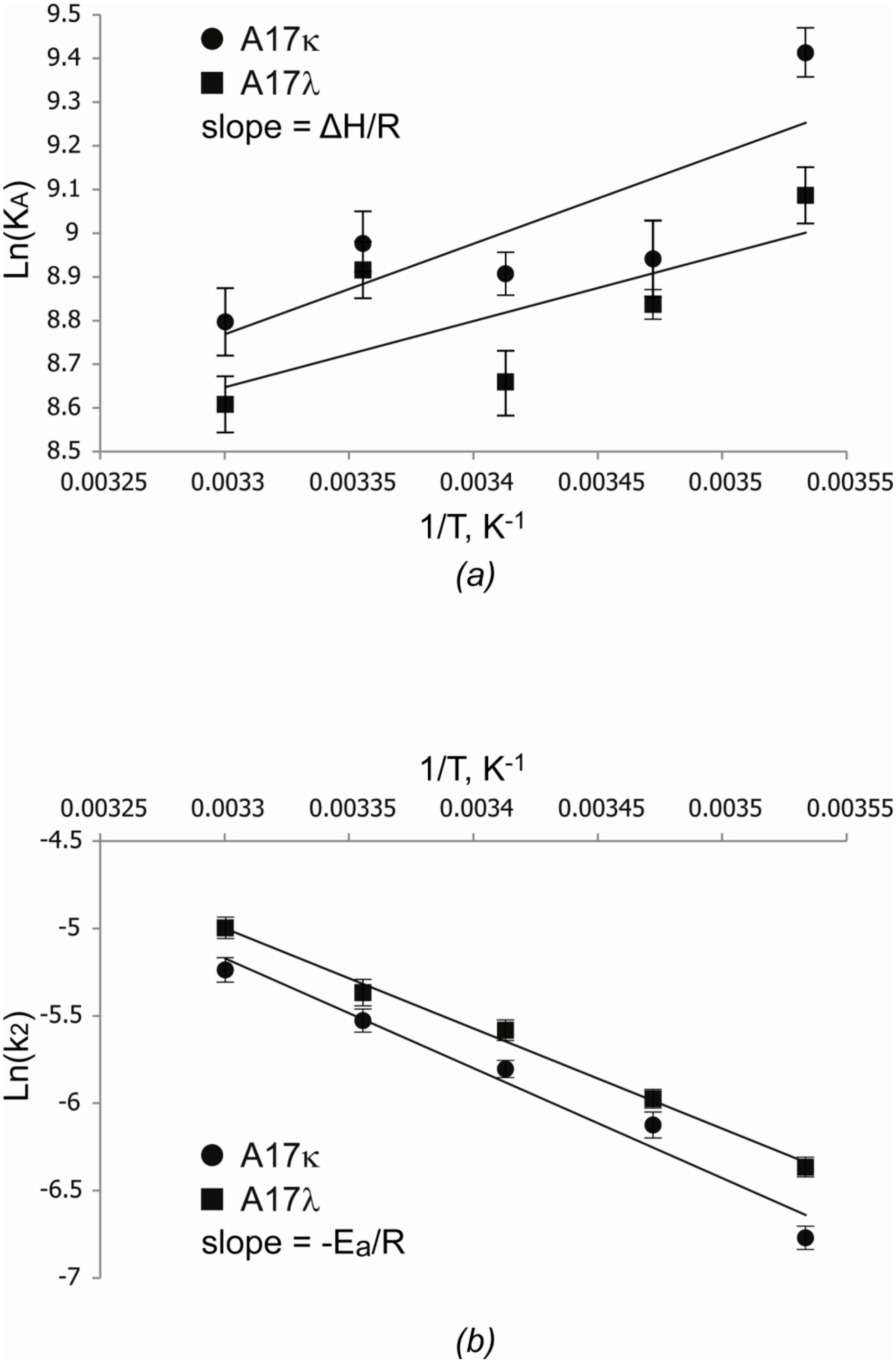

Figure S7. (a) Van't Hoff and (b) Arrhenius analysis of the temperature dependence of phosphonate X interactions with A17κ and A17λ reactibodies.

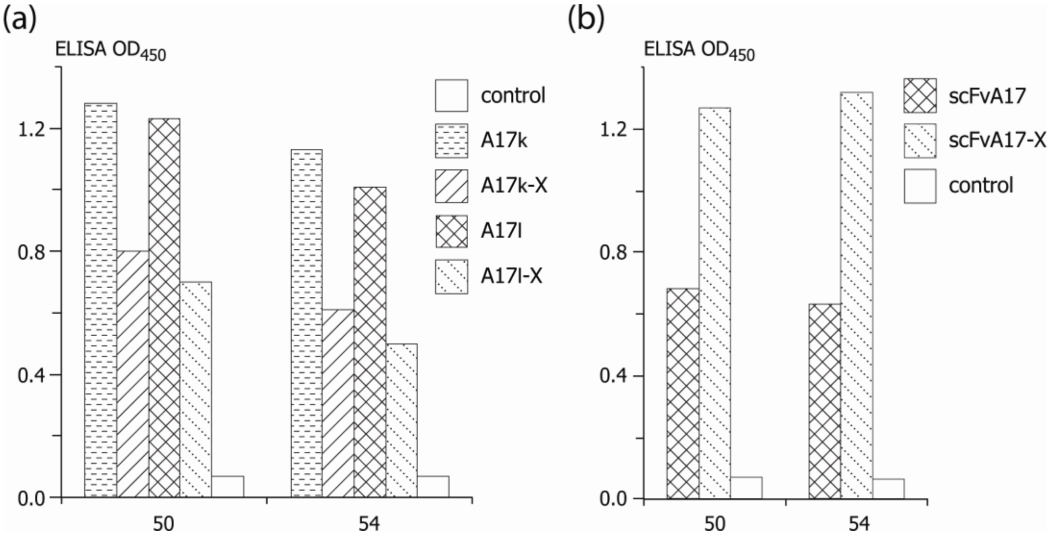

Figure S8. Interactions of (a) Fabs of A17κ and A17λ, and (b) scFvA17 and their variants modified by phosphonate X with phage-displayed cyclic peptides 50C and 54C as measured by ELISA. Recombinant human antibody was used as a negative control.

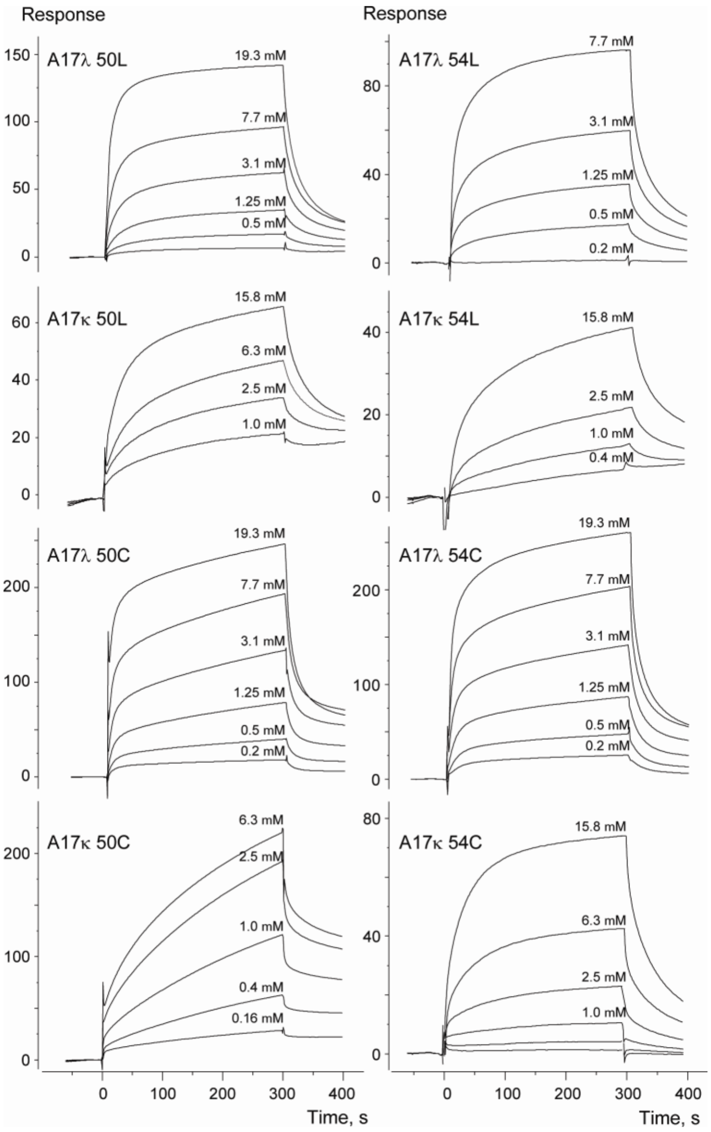

Figure S9. BiaCore sensorgrams and affinity analysis of interactions of linear (50L, 54L) and cyclic peptides (50C, 54C) with A17κ and A17λ reactibodies.

Supporting references

Koivunen, E., Wang, B. & Ruoslahti, E. (1994). *J Cell Biol* **124**, 373-380.

Parmley, S. F. & Smith, G. P. (1988). *Gene* **73**, 305-318.

Reshetnyak, A. V., Armentano, M. F., Ponomarenko, N. A., Vizzuso, D., Durova, O. M., Ziganshin, R., Serebryakova, M., Govorun, V., Gololobov, G., Morse, H. C., 3rd, Friboulet, A., Makker, S. P., Gabibov, A. G. & Tramontano, A. (2007). *J Am Chem Soc* **129**, 16175-16182.

Yribarren, A. S., Thomas, D., Friboulet, A. & Avalle, B. (2003). *Eur J Biochem* **270**, 2789-2795.
